# Conserved 4-coumarate 3-hydroxylase/ascorbate peroxidase bifunctionality coordinates lignin deposition and plant growth in *Brachypodium* and *Populus*

**DOI:** 10.1093/plphys/kiag293

**Published:** 2026-05-22

**Authors:** Weiwei Zhu, Harmeet Singh-Bakala, Bo Liu, William Bewg, Max Bentelspacher, Rachel Weber, María Ángeles Peláez-Vico, Margot S S Chen, Chung-Jui Tsai, Ron Mittler, Bing Yang, Jaime Barros

**Affiliations:** Division of Plant Science and Technology, College of Agriculture Food and Natural Resources and Interdisciplinary Plant Group, University of Missouri, Columbia, MO 65211, United States; Division of Plant Science and Technology, College of Agriculture Food and Natural Resources and Interdisciplinary Plant Group, University of Missouri, Columbia, MO 65211, United States; Division of Plant Science and Technology, College of Agriculture Food and Natural Resources and Interdisciplinary Plant Group, University of Missouri, Columbia, MO 65211, United States; School of Forestry and Natural Resources, University of Georgia, Athens, GA 30602, United States; Center for Bioenergy Innovation (CBI), Oak Ridge National Laboratory, Oak Ridge, TN 37830, United States; Division of Plant Science and Technology, College of Agriculture Food and Natural Resources and Interdisciplinary Plant Group, University of Missouri, Columbia, MO 65211, United States; Division of Plant Science and Technology, College of Agriculture Food and Natural Resources and Interdisciplinary Plant Group, University of Missouri, Columbia, MO 65211, United States; Division of Plant Science and Technology, College of Agriculture Food and Natural Resources and Interdisciplinary Plant Group, University of Missouri, Columbia, MO 65211, United States; Christopher S. Bond Life Sciences Center, University of Missouri, Columbia, MO 65211 United States; School of Forestry and Natural Resources, University of Georgia, Athens, GA 30602, United States; Center for Bioenergy Innovation (CBI), Oak Ridge National Laboratory, Oak Ridge, TN 37830, United States; School of Forestry and Natural Resources, University of Georgia, Athens, GA 30602, United States; Center for Bioenergy Innovation (CBI), Oak Ridge National Laboratory, Oak Ridge, TN 37830, United States; Department of Plant Biology, University of Georgia, Athens, GA 30602, United States; Department of Genetics, University of Georgia, Athens, GA 30602, United States; Division of Plant Science and Technology, College of Agriculture Food and Natural Resources and Interdisciplinary Plant Group, University of Missouri, Columbia, MO 65211, United States; Christopher S. Bond Life Sciences Center, University of Missouri, Columbia, MO 65211 United States; Division of Plant Science and Technology, College of Agriculture Food and Natural Resources and Interdisciplinary Plant Group, University of Missouri, Columbia, MO 65211, United States; Donald Danforth Plant Science Center, St.Louis, MO 63132, United States; Division of Plant Science and Technology, College of Agriculture Food and Natural Resources and Interdisciplinary Plant Group, University of Missouri, Columbia, MO 65211, United States

## Abstract

Cytosolic ascorbate peroxidases (APXs) have been proposed to have bifunctional 4-coumarate 3-hydroxylase (C3H) activity, linking redox regulation to lignin biosynthesis in plants. Although this dual role has been shown in vitro, in vivo validation remains limited. Here, we used CRISPR/Cas9 gene editing to knock out cytosolic C3H/APX genes in *Brachypodium distachyon* and poplar (*Populus tremula × Populus alba*). In *Brachypodium*, BdC3H/APX1 catalyzed the ascorbate-dependent hydroxylation of 4-coumarate to caffeate in vitro. Loss of BdC3H/APX1 function led to reduced lignin content, altered monomer composition, elevated H_2_O_2_ levels, and impaired growth, while double monoallelic knockouts of *BdC3H/APX1* combined with a biallelic *BdC3H/APX2* mutation (*Bdc3h/apx1&2*) exhibited severe developmental defects. Exogenous caffeate and ferulate rescued the growth and lignin phenotype of the *Bdc3h/apx1* knockout mutants, whereas catalase reduced H_2_O_2_ without restoring plant growth. Similarly, CRISPR/Cas9-mediated *PtC3H/APX1* knockout in poplar resulted in stunted growth and altered lignin composition, while the double *Ptc3h/apx1&2* mutants were unable to regenerate from tissue culture. These results provide in vivo evidence of C3H/APX bifunctionality, suggesting that perturbed lignin biosynthesis is the primary cause of the growth defects typically observed in C3H/APX-deficient plants.

## Teaser

Cytosolic bifunctional 4-coumarate 3-hydroxylase/ascorbate peroxidase activity links oxidative stress to lignin biosynthesis and is essential for normal plant growth in model grass and woody dicot species.

## Introduction

Lignin is a naturally abundant phenolic polymer found in plant cell walls that provides structural support and is essential for plant upright growth. Lignin also plays a crucial role in water transport by reinforcing xylem vessels (XV), thereby facilitating efficient water and nutrient assimilation (Boyce et al. 2004; Reyt et al. 2021). Additionally, it offers protection as a defense mechanism against pathogens and other environmental stresses (Cesarino 2019; Ménard et al. 2022; Yadav and Chattopadhyay 2023; Zhu et al. 2025). Research on lignin biosynthesis is relevant not only for understanding plant biology, but also for its potential as a renewable resource for the production of bio-based materials, biofuels, and commodity chemicals (Ragauskas et al. 2014; Liu et al. 2022; Dixon et al. 2024; Zhu and Barros 2025).

Lignin biosynthesis involves a series of enzymatic reactions primarily occurring in the cytosol and apoplast of plant cells. In the cytosol, the phenylpropanoid pathway synthesizes monolignols: *p*-coumaryl alcohol, coniferyl alcohol, and sinapyl alcohol (Dixon and Barros 2019; Vanholme et al. 2019). Monolignols are then polymerized in the apoplast into 3 major lignin subunits: *p*-hydroxyphenyl or H-units, guaiacyl or G-units, and syringyl or S-units, via oxidative radical coupling reactions catalyzed by secretory laccases (LACs) and Class III peroxidases (PRXs; Zhao et al. 2013; Tobimatsu and Schuetz 2019; Blaschek and Pesquet 2021). These oxidative enzymes, collectively known as phenoloxidases, mediate the extracellular polymerization of monolignols into lignin, shaping the functional properties of plant cell walls. Redox reactions are key during lignification, where hydrogen peroxide (H_2_O_2_) and molecular oxygen (O_2_) are substrates for PRXs and LACs, respectively, enabling the oxidative polymerization of monolignols (Barceló 1998; Onnerud et al. 2002; Warinowski et al. 2016). Therefore, maintaining overall redox balance, particularly the availability of H_2_O_2_, is crucial for proper lignin assembly in plant cell walls.

In contrast to typical Class III secretory PRX, which have been reported to be involved in lignin polymerization in the apoplast (Marjamaa et al. 2009; Tobimatsu and Schuetz 2019; Rojas-Murcia et al. 2020), Class I ascorbate peroxidases (APXs) function mainly in the cytosol, where they use ascorbic acid (AsA) as electron donor to catalyze the conversion of H_2_O_2_ into water to maintain redox balance and protect cells against oxidative stress (Mittler and Poulos 2005; Bonifacio et al. 2011; Pandey et al. 2017b; Yoshimura et al. 2024). Therefore, APXs are constitutively expressed across many plant tissues, including roots, leaves, and stems and are strongly upregulated in response to a range of biotic and abiotic stresses (Zimmermann and Zentgraf 2005; Zhang et al. 2022; Pang et al. 2023; Singh et al. 2024). Cytosolic APX was first genetically identified in pea (*Pisum sativum*) as an H_2_O_2_-scavenging enzyme (Mittler and Zilinskas 1992). In *Arabidopsis thaliana*, loss-of-function of cytosolic APX1 has been shown to result in growth inhibition, delayed flowering, and elevated H_2_O_2_ levels under normal growth conditions (Pnueli et al. 2003; Koussevitzky et al. 2008). Similarly, transgenic rice silenced through RNA interfering (RNAi) for cytosolic APX1 displayed a semi-dwarf phenotype and increased H_2_O_2_ accumulation (Rosa et al. 2010). APX1 has been shown to be essential for protecting the thylakoid membrane and nuclear DNA from light-induced oxidative damage (Davletova et al. 2005; Vanderauwera et al. 2011), and deficiency in APX2 resulted in a decreased tolerance to light stress, as well as an enhanced tolerance to salinity and oxidative stresses (Suzuki et al. 2013). While some evidence suggests that H_2_O_2_ overaccumulation may alter the expression of transcription factors involved in plant growth regulation (Pnueli et al. 2003; Tian et al. 2018; Khedia et al. 2019), further studies are required to identify the direct cause of the developmental defects observed in APX-deficient plants.

In addition to their established role as PRXs in reactive oxygen species (ROS) metabolism and oxidative stress responses, APXs are also involved in other biochemical pathways (Li, 2023). For instance, a mitochondrial APX from *Populus tomentosa* (PtomtAPX), has been reported to relocate to the cell wall after programmed cell death, where it functions not only in detoxifying H_2_O_2_ but also in oxidizing monolignols during the early stages of lignification (Zhang et al. 2022). While PtomtAPX oxidizes all 3 monolignols with a preference for sinapyl alcohol, its catalytic efficiency (*k*_cat_/*K*_m_) for monolignols is over 100-fold lower than that for AsA (Zhang et al. 2022). During early development (1 to 2 mo), PtomtAPX-knockdown plants were shorter and exhibited thinner secondary walls and reduced lignin content, particularly in S-lignin subunits. PtomtAPX expression declines sharply at 3 mo of age in wild-type (WT) plants, and lignification in PtomtAPX-knockdown stems is comparable to WT at this stage, suggesting that Class III PRXs and LACs may assume the role of lignin polymerization in later developmental stages. Similarly, cytosolic APX1 isoforms from *Sorghum bicolor* (SbAPX) and *Panicum virgatum* (PvAPX) catalyze the oxidative polymerization of a broad range of monolignol pathway intermediates, including phenolic acid and aldehydes, using H_2_O_2_ as a substrate (Zhang et al. 2023a, 2023b). The potential translocation of SbAPX and PvAPX to the cell wall and genetic evidence from loss-of-function mutants to support their physiological role in lignification has yet to be reported. Nevertheless, these findings suggest that the role of APXs in lignification may be more extensive than previously recognized.

Unlike chloroplastic APX isoforms, cytosolic APX has been shown to oxidize a broad range of aromatic compounds, including guaiacol, *p*-cresol, *o*-dianisidine, and ABTS at rates comparable to ascorbate (Heering et al. 2001; Shigeoka et al. 2002; Raven et al. 2004). We previously identified a cytosolic APX in maize with bifunctional 4-coumarate 3-hydroxylase activity (C3H/APX), which provides an alternative route for the direct conversion of 4-coumarate to caffeate in the early steps of lignin biosynthesis pathway (Barros et al. 2019). Biochemical and genetic evidence in *Brachypodium distachyon* and *A. thaliana* showed that C3H/APX uses AsA as a reducing agent to hydroxylate 4-coumarate, thereby linking redox metabolism to monolignol biosynthesis through a single enzymatic step. Brachypodium *c3h/apx1*-knockdown lines exhibited a reduced lignin content, accompanied with a stunted growth phenotype, increased proportion of non-viable seeds, and delayed senescence. Further multi-omics characterization of these lines revealed a global shift in proteome and metabolite profiles, including altered abundance of lignin biosynthetic enzymes and disrupted redox homeostasis (Shrestha et al. 2022). However, it remains unclear whether the growth inhibition of *c3h/apx1*-knockdown lines results from redox imbalance, impaired lignin biosynthesis, a combination of these, or additional factors. Moreover, C3H/APXs function has not yet been examined using CRISPR/Cas9 gene editing, which offers a more precise approach to achieve loss of function mutations compared with the RNAi and T-DNA insertion lines reported to date (Pnueli et al. 2003; Rosa et al. 2010; Suzuki et al. 2013; Barros et al. 2019; Shrestha et al. 2022).

In this study, we used CRISPR/Cas9 to generate single and double C3H/APX-knockout plants in the model monocot grass *B. distachyon* and in dicot hybrid poplar trees (*Populus tremula* × *Populus alba* INRA 717-1B4). Phenotypic analyses revealed that loss of C3H/APX function leads to altered lignin deposition, elevated H_2_O_2_ levels, and severe developmental defects in both plant species. Exogenous caffeate (Ex-CAF) and ferulate feeding rescued lignin and growth defects in *c3h/apx*-deficient Brachypodium, whereas catalase (CAT) treatments reduced H_2_O_2_ but failed to restore growth, suggesting that altered lignin biosynthesis contributes to the observed developmental defects. Taken together, our findings highlight the dual role of C3H/APXs in both redox homeostasis and monolignol biosynthesis during plant development, with implication for engineering bioenergy crops for biomass yield and stress tolerance.

## Results

### Selection of C3H/APX homologs for functional characterization

Plant PRXs are divided into 2 families, Class I PRXs of prokaryotic origin and Class III plant secretory PRXs (Fig. S1a). C3H/APXs belong to the Class I type and are conserved in bacteria, fungi and both monocot and dicot plants. In *A. thaliana*, the C3H/APX family members are localized in the cytosol, mitochondria, chloroplasts, and peroxisomes (Mittler and Poulos 2005; Lazzarotto et al. 2021). Using the amino acid sequence of the cytosolic *A. thaliana* AtC3H/APX1 (AT1G07890) as a reference, our phylogenetic analysis identified 2 cytosolic C3H/APXs homologs in *B. distachyon* Bradi1g65820 (BdC3H/APX1) and Bradi1g16510 (BdC3H/APX2), sharing 78% and 79% identity with AtC3H/APX1, respectively (Fig. S1a). Based on previous work (Shrestha et al. 2022), both proteins are abundant in *B. distachyon* stems, with BdC3H/APX1, which encodes 2 protein isoforms (I1H6P1 and I1H6P2) showing slightly higher protein levels than BdC3H/APX2 (Fig. S1b). In the dicot clade, we identified 3 homologs in *Populus trichocarpa*: Potri.009G015400 (PtC3H/APX1), Potri.016G084800 (PtC3H/APX2), and Potri.006G132200 (PtC3H/APX3), each sharing 81%, 80%, and 80% sequence identity to *A. thaliana* C3H/APX1 (Fig. S1a). We targeted *PtC3H/APX1* and *PtC3H/APX2* due to their higher gene expression levels in poplar xylem tissues (Fig. S1c). Functional characterization was conducted in *B. distachyon* inbred line Bd21-3 and the genetically tractable poplar genotype *P. tremula* × *P. alba* INRA 717-1B4, as model monocot and woody dicot systems, respectively (Fig. S1d).

### C3H activity of *Bd*C3H/APX requires ascorbate and reconstitution with hemin

**Figure 1:**
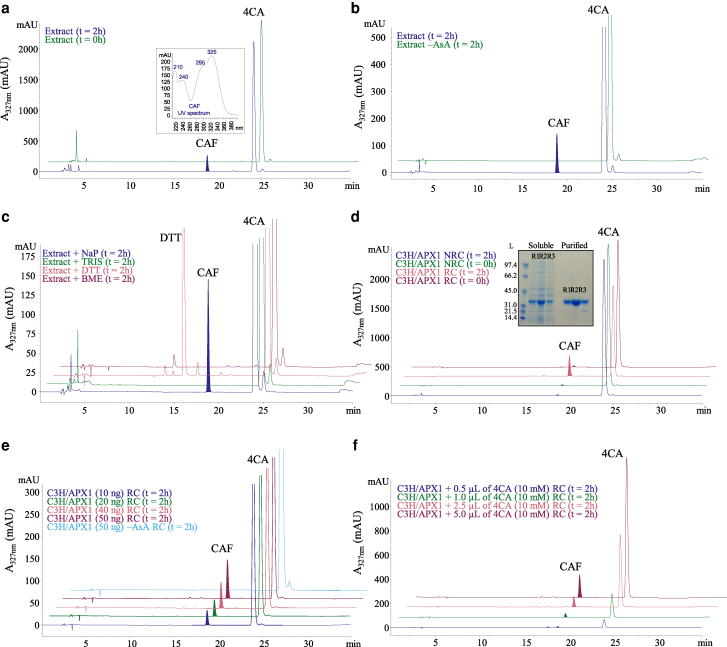
Enzymatic activity of C3H/APX measured by HPLC. a) C3H activity assay using crude protein extracts from WT *Brachypodium* stem tissues. b) Comparison of enzyme activities with and without ascorbate. c) Comparison of enzyme reaction containing 50 mm phosphate buffer, pH = 6.0 (NaP) and 50 mm Tris–HCl, pH 7.5 (TRIS) buffer and reducing agents DTT and BME. d) In vitro C3H/APX activity assays with non-reconstituted (NRC) and hemin-reconstituted (RC) recombinant BdC3H/APX1 purified protein. The SDS-PAGE gel of 3 soluble and purified fractions of recombinant BdC3H/APX protein is shown. e) In vitro C3H/APX activity assays with increased concentrations of recombinant protein. f) In vitro C3H/APX activity assays with increased concentration of substrate, 4CA. 4CA, 4-coumaric acid; CAF, caffeic acid. Crude extract assay: Reaction (100 *μ*L total) contains 10 *μ*L 10 mm 4CA, 7.5 *μ*L 10 mg/mL BSA, 20 *μ*L 20 mM l-ascorbate, 7.5 *μ*L 1 M sodium phosphate buffer (pH 6.0), 15 *μ*L *Brachypodium* stem crude extract (0.7 *μ*g/*μ*L), and water. Recombinant BdC3H/APX1 assay: Reaction (100 *μ*L total) contains 10 *μ*L 10 mm 4CA, 7.5 *μ*L 10 mg/mL BSA, 20 *μ*L 20 mM l-ascorbate, 7.5 *μ*L 1 M phosphate buffer (pH 6.0), 10 *μ*L 0.3% H_2_O_2_, 500 ng hemin-reconstituted (RC) or NRC purified BdC3H/APX1, and water. *t* = 0 h, reaction quenched immediately with 10 *μ*L acetic acid; *t* = 2 h, incubated at 30 °C for 2 h then quenched. The Michaelis–Menten kinetics of BdC3H/APX1 were previously reported (Barros et al. 2019).

Our previous work identified C3H/APXs in *B. distachyon* and reported its C3H activity and kinetic properties (Barros et al. 2019). However, recent studies using C3H/APX homologs in sorghum and switchgrass reported that the conversion of 4-coumaric acid (4CA) to caffeic acid (CAF) in the presence of H_2_O_2_ and AsA can occur via a non-enzymatic reaction (Zhang et al. 2023a, 2023b). To re-evaluate the enzymatic activity of BdC3H/APX, we prepared crude protein extracts from WT *B. distachyon* stems and performed in vitro enzyme activity assays. Reactions containing 4CA, AsA, and the crude protein extract were analyzed by HPLC (Fig. 1a). A peak corresponding to CAF was detected after 2 h incubation (*t* = 2 h) but was absent in control reactions quenched with glacial acetic at the start of the incubation (*t* = 0 h). The retention time and UV absorbance spectrum at 327 nm of the product matched that of an authentic CAF standard (inset Fig. 1a). This peak of CAF was only observed in the presence of AsA, indicating that AsA is required as a reducing agent for the C3H/APX reaction (Fig. 1b). Moreover, the substitution of sodium phosphate buffer (pH 6.0) with Tris buffer (pH 7.5), or the addition of reducing agents dithiothreitol (DTT) and β-mercaptoethanol (BME), completely depleted the C3H activity (Fig. 1c). Using ∼10 *µ*g of crude protein extract, the formation of CAF plateaued at around 600 *µ*m of 4CA and remained steady up to 2,000 *µ*m (Fig. S2a to c). Additionally, the AsA peak areas (measured at 280 nm) decreased in the incubated sample compared with the background reaction (*t* = 0 h), indicating that AsA is used in the C3H reaction as a reducing agent (Fig. S2d). Note that the C3H reaction using crude protein extracts differs from that of the purified enzyme in that no exogenous H_2_O_2_ was added, as we previously observed that AsA is rapidly consumed if H_2_O_2_ is supplemented to the crude extract assays (Barros et al. 2019). Overall, these results show that the conversion of 4CA to CAF is enzymatic and support C3H/APX activity in the *B. distachyon* crude protein extract.

Recombinant BdC3H/APX1 (Bradi1g65820) was expressed in *Escherichia coli* and purified for in vitro analysis of its C3H activity. Our results show that effective conversion of 4CA into CAF requires the hemin-reconstituted form of the BdC3H/APX1 enzyme, whereas the non-heme-constructed reaction did not produce CAF, providing a negative control and arguing against non-enzymatic CAF formation in our reaction mixture (Fig. 1d). Increasing amounts of recombinant BdC3H/APX1 (10 to 50 ng) led to higher CAF peaks, and no product was detected in the absence of AsA, showing that the C3H activity depends on the presence of AsA (Fig. 1e). Furthermore, reactions containing a constant amount of enzyme and increasing 4CA concentrations (50 to 500 *µ*m) yielded proportionally higher CAF peaks, indicating that product formation increases with substrate availability (Fig. 1f). These data indicate that BdC3H/APX1 catalyzes the enzymatic 3-hydroxylation of 4CA to CAF using AsA as electron donor, with both the 4CA substrate concentration and the amount of enzyme directly influencing the CAF product yield. The standard Michaelis–Menten kinetics of BdC3H/APX1 have been described previously (Barros et al. 2019).

### Disruption of *BdC3H/APXs* leads to developmental defects in *Brachypodium*

**Figure 2:**
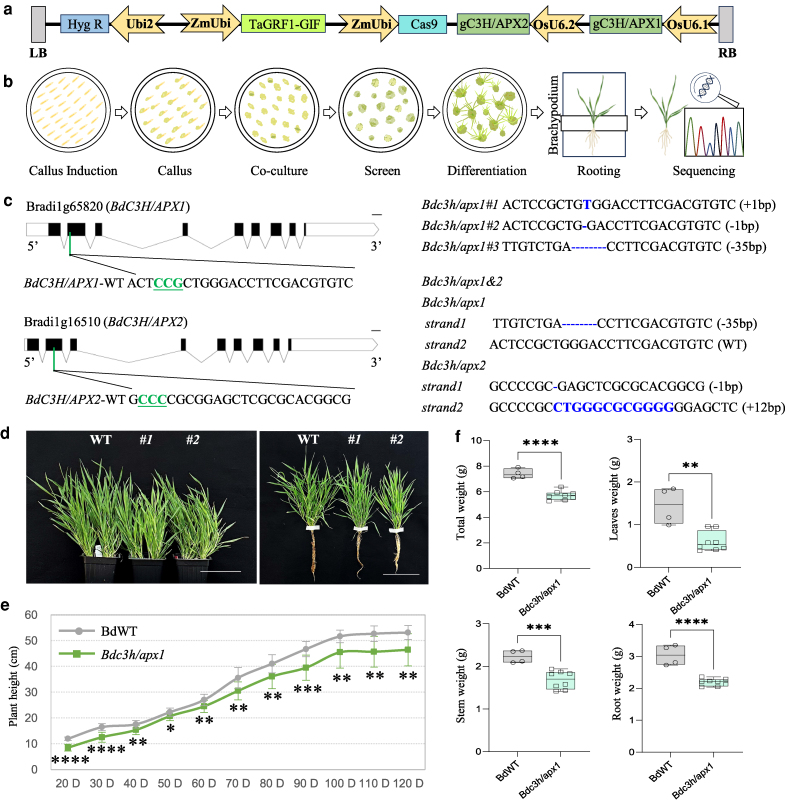
CRISPR/Cas9 gene editing and developmental phenotype of *Bdc3h/apx1* in *B. distachyon.* a) Structure of CRISPR/Cas9 vector for *BdC3H/APXs* genes. LB, left border; Ubi2, ubiquitin 2 promoter; Hyg R, hygromycin resistance gene; ZmUbi, *Zea mays* ubiquitin promoter; TaGRF1-GIF, chimeric protein from *Triticum aestivum* growth regulating factor1 (GRF1) and GRF-interacting factor (GIF) to enhance plant regeneration efficiency; Cas9, DNA sequence of Cas9; OsU6.1 and OsU6.2, promoter to drive gRNAs expression; gC3H/APX2, guide RNA of BdC3H/APX2; gC3H/APX1, guide RNA of BdC3H/APX2; RB, right border. b) Schematic diagram of the *BdC3H/APXs* knockout transgenic *Brachypodium* plant generation process. Callus formation was initiated using mature seeds collected from ∼100-d-old plants. c) Target sequences of *BdC3H/APX* genes. Underlined letters indicate the PAM site; blue shows the edited region. d) Growth phenotype of *Bdc3h/apx1* plants 60 d after planting (DAP). Scale bar = 10 cm. e) Changes in plant height at different developmental stages (*n* > 15 plants). D, days after planting. f) Biomass of Brachypodium WT and *Bdc3h/apx1* mutant plants, including fresh weights of total biomass, leaves, stems, and roots at ∼9 wk of growth (60 DAP). Box plots indicate the median (center lines), interquartile range (hinges), and whiskers represents min and max values. Error bars indicate mean ± Sd. Statistical analysis was performed using 2-sided unpaired *t*-tests: *P < 0.05; **P < 0.01; ***P < 0.001; ****P < 0.0001.

To investigate the functional roles of *C3H/APX* genes in *B. distachyon*, we generated CRISPR-Cas9-edited mutants targeting both *BdC3H/APX1* and *BdC3H/APX2* with single guide RNAs (sgRNA) designed to target the second exon of each gene (Figs. 2a to c and S3a). During plant transformation, callus induction was achieved directly from mature seeds, eliminating the need for continuous plant propagation to obtain fresh seed and significantly reducing the overall transformation timeline (Fig. 2b). T_0_ plants with confirmed gene edits were propagated to obtain T_1_ lines. We identified 3 independent *BdC3H/APX1* mutant lines, each carrying homozygous frameshift mutations: *Bdc3h/apx1#1* (+1 bp insertion), *Bdc3h/apx1#2* (−1 bp deletion), and *Bdc3h/apx1#3* (−35 bp deletion) at the target site (Figs. 2c and S3b). In addition, we obtained a double *Bdc3h/apx1&2* mutant plant with a monoallelic 35 bp deletion in *BdC3H/APX1* (−35 bp/WT) and biallelic deletions in *BdC3H/APX2* (−1 bp/+12 bp) (Figs. 2c and S3c). Unfortunately, we did not recover any *BdC3H/APX2* knockout mutants (Fig. S3). Homozygous T_2_ generation plants were selected for further phenotypic and biochemical characterization. Phenotypic analyses revealed that *Bdc3h/apx1* single mutants exhibited significantly reduced plant height at both vegetative (60 DAP, days after planting) and reproductive (90 DAP) stages compared with WT controls (Figs. 2d and e and S4a and b). These mutants also displayed reduced biomass in leaves, stems and roots (Fig. 2f). These growth defects were more pronounced in the *Bdc3h/apx1&2* double mutants, which showed a severe developmental delay, along with defective flower development and seed production (Fig. S4c and d). These results indicate that *BdC3H/APX* genes contribute to normal plant development, and suggest that simultaneous disruption of both *BdC3H/APX1* and *BdC3H/APX2* leads to additive defects in plant growth, flowering, and seed production in *Brachypodium*.

### Loss-of-function of *BdC3H/APXs* alters overall lignin content and monolignol composition in *Brachypodium*

**Figure 3:**
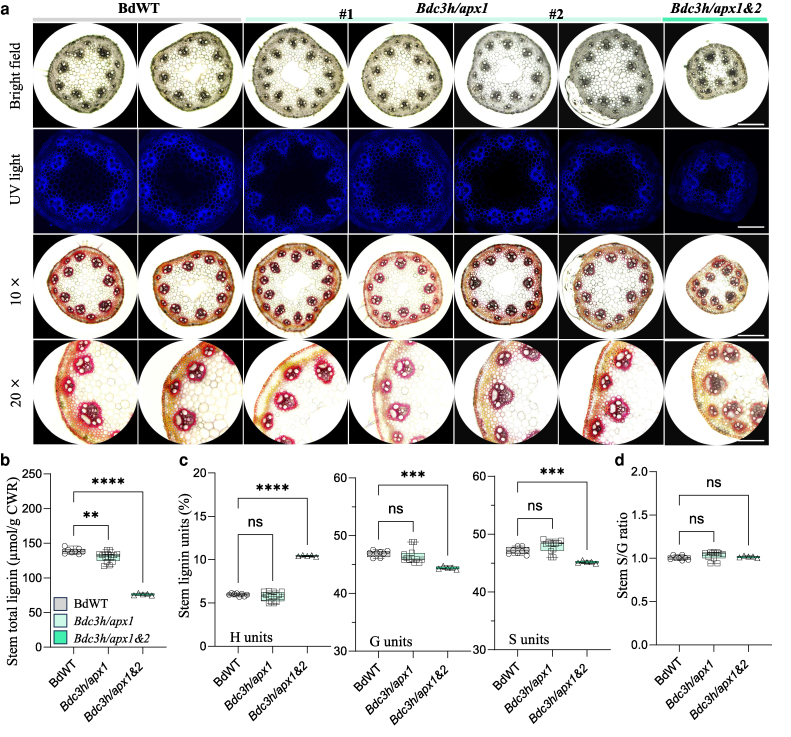
Lignin phenotype of *Bdc3h/apx* mutant plants at 60 d after planting. a) Representative stem cross-sections of WT (BdWT), *Bdc3h/apx1* (#1 and #2), and *Bdc3h/apx1&2* mutant plants visualized under bright field (top row), UV autofluorescence (second row), and stained with phloroglucinol-HCl under 10× and 20× magnification (third and fourth rows). Scale bars: 370 *μ*m for all brightfield and 10× images, and 180 *μ*m for all UV and 20× images. b) Quantification of total lignin content in stems using the acetyl bromide method. c) Quantification of thioacidolysis-released H-, G-, and S-lignin monomers in stem CWRs. d) S/G ratio in stem lignin of mutant and WT plants (*n* > 5). Box plots indicate the median (center lines), interquartile range (hinges), and whiskers represents min and max values. Data points for all biological replicates are shown. Statistical analysis was performed using 1-way ANOVA followed by Dunnett's test for multiple comparisons: **P < 0.01; ***P < 0.001; ****P < 0.0001; ns, not significant.

To determine whether *BdC3H/APX* genes influence lignin deposition, we examined stem cross-sections of *Bdc3h/apx1* and *Bdc3h/apx1&2* mutants using UV-microscopy and phloroglucinol-HCl staining. Compared with WT plants, both single and double mutants exhibited lighter lignin autofluorescence and less intense phloroglucinol-HCl staining in interfascicular fibers and vascular bundles, with a strong reduction observed in *Bdc3h/apx1&2* plants (Fig. 3a). To quantify these differences, we measured the lignin content and monomer composition in the *Bdc3h/apx1* and *Bdc3h/apx1&2* plants using thioacidolysis followed by GC-MS. Both *Bdc3h/apx1* and *Bdc3h/apx1&2* mutants showed significantly reduced lignin levels, with the double mutant exhibiting a ∼50% decrease compared with WT (Fig. 3b). Additionally, lignin compositional analysis of *Bdc3h/apx1&2* stems revealed a significant increase in the proportion of H-units, accompanied by reductions in both guaiacyl (G) and syringyl (S) units, with no significant changes in the S/G lignin monomer ratio (Fig. 3c and d). Plants after flowering (90 DAP) showed a similar lignin phenotype, with reduced total lignin content and no significant change in the S/G ratio (Fig. S5). Together, these results show that cytosolic *BdC3H/APXs* contribute to the lignification of vascular tissues in *B. distachyon*, and that simultaneous disruption of both genes has an additive effect on lignin biosynthesis.

### *PtC3H/APXs* disruption causes similar growth and lignin defects in *Populus*

**Figure 4:**
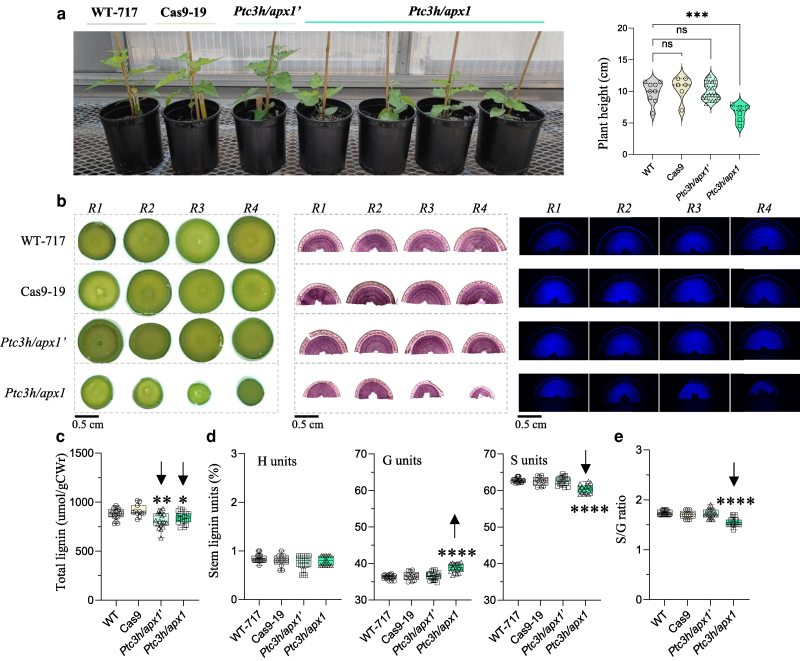
Growth and lignin phenotype of *Ptc3h/apx* in poplar (*P. tremula × P. alba INRA* 717-1B4). a) Growth phenotype of WT, Cas9-only control, *Ptc3h/apx1′* and *Ptc3h/apx1* plants and violin plot of plant height. *Ptc3h/apx1′* are heterozygous mutants (−/+), including 3 indel patterns: −1 bp/WT, −30 bp/WT and +1 bp/WT. *Ptc3h/apx1* are homozygous mutants (−/−), including 2 indel patterns: NA/−17 bp and +1 bp/−1 bp. NA, no-amplification, suggesting large dropouts. b) Transverse stem sections showing cross-sectional area (left), phloroglucinol-HCl staining for lignin (middle), and UV autofluorescence (right) of vascular tissue from the genotypes shown in (a). Scale bars: 180 *μ*m. c) Total lignin content in stem tissues measured by thioacidolysis followed by GC-MS. d) Relative abundance of H-, G-, and S-lignin monomers as a percentage of total monomers in the same tissues. e) Syringyl/guaiacyl (S/G) ratio in stems tissues. Box plots indicate the median (center lines), interquartile range (hinges), and whiskers represents min and max values. Error bars indicate mean ± Sd. Data points for all biological replicates are shown (*n* > 10). Statistical analysis was performed using 1-way ANOVA followed by Dunnett's test for multiple comparisons: *P < 0.05; **P < 0.01; ***P < 0.001; ****P < 0.0001; ns, not significant.

To investigate whether *C3H/APX* genes play conserved roles in lignin biosynthesis and plant development in a woody dicot species, we generated CRISPR/Cas9-edited lines targeting *PtaC3H/APX1* and *PtaC3H/APX2* in hybrid poplar trees (*P. tremula × P. alba* INRA 717-1B4). A series of heterozygous (*Ptc3h/apx1′*, −1 bp/WT, −30 bp/WT and +1 bp/WT) and homozygous (*Ptc3h/apx1*, NA/−17 bp and +1 bp/−1 bp) mutant lines were obtained (Tables S1 and S2). Phenotypic analysis revealed that the *Ptc3h/apx1′* heterozygous mutants were indistinguishable from WT plants, whereas *Ptc3h/apx1* homozygous mutants exhibited significantly reduced plant height and stem diameter (Fig. 4a and b). Stem cross-sections from *Ptc3h/apx1* mutant and WT plants were examined under UV light and stained with phloroglucinol-HCl to assess lignin deposition. Both assays revealed reduced lignin deposition in *Ptc3h/apx1* stems, with reduced lignin autofluorescence and staining intensity compared with control plants (Fig. 4b). Thioacidolysis analysis revealed a significant reduction in total lignin content in both *Ptc3h/apx1′* and *Ptc3h/apx1* lines (Fig. 4c). However, only the biallelic *Ptc3h/apx1* mutant lines displayed reduced S/G lignin monomer ratios, with increased G-lignin and decreased S-lignin levels (Fig. 4d and e). These findings indicate that *PtC3H/APX1* contributes to lignin biosynthesis and modulates monolignol composition in poplar. Despite multiple transformation attempts, we were unable to obtain double *PtC3H/APX1&2* knockout plants in poplar. Notably, double-gene knockout calli were generated but could not induce shoot formation (Fig. S6), suggesting that simultaneous loss of both *PtC3H/APX1&2* genes, or the *PtC3H/APX2* gene alone, is detrimental to organogenesis in *Populus*. Taken together, these results indicate that *C3H/APX* genes exhibit comparable phenotypes in monocot and dicot species, contributing to normal plant growth and lignin deposition.

### Loss of *BdC3H/APX* function leads to H_2_O_2_ overaccumulation in *Brachypodium*

**Figure 5:**
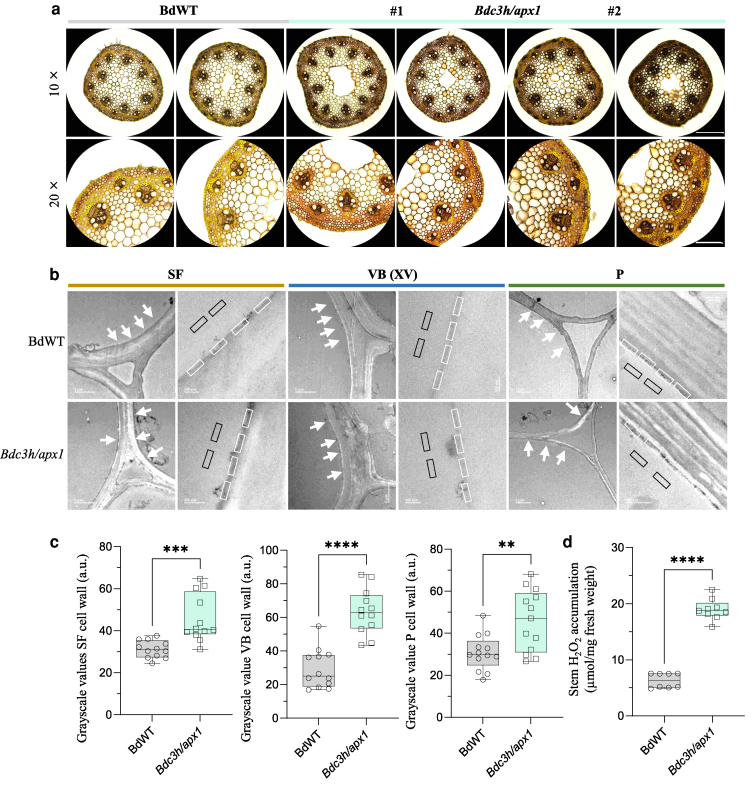
H_2_O_2_ accumulation in *Bdc3h/apx1* mutants. a) DAB staining of stem cross-sections from 60-d-old *Bdc3h/apx1* and WT (BdWT) plants. Sections were imaged under brightfield to visualize H_2_O_2_ accumulation (brown precipitate). Scale bars: 370 *μ*m for all 10× images, and 180 *μ*m for all 20× images. Several of the stem tissue sections shown in (a) are the same as those shown in Fig. 3a. b) TEM of SF, vascular bundles (VB/XV), and parenchyma cells (P) following cerium chloride (CeCl_3_) staining to detect H_2_O_2_ localization in situ. White arrows indicate cerium perhydroxide precipitates at the plasma membrane-cell wall interface (see Fig. S8 for details). White and black squares indicate the areas where we obtained the values for the H_2_O_2_ precipitates and background controls, respectively. c) Grayscale intensity from TEM micrographs showing increased cerium deposits in the cell walls of SF, VB, and P cells in *Bdc3h/apx1* mutants. d) Biochemical quantification of H_2_O_2_ accumulation in stem tissues using the Amplex Red assay (*n* > 8). Box plots indicate the median (center lines), interquartile range (hinges), and whiskers represents min and max values. Error bars indicate mean ± Sd. Data points for all biological replicates are shown. Statistical analysis was performed using 1-way ANOVA followed by Dunnett’s test for multiple comparisons: **P < 0.01; ****P < 0.0001.

APXs are antioxidant enzymes that scavenge H_2_O_2_ using AsA as electron donor (Shigeoka et al. 2002; Caverzan et al. 2012; Li, 2023). To assess whether *BdC3H/APX* influences cellular redox homeostasis, we examined H_2_O_2_ levels using multiple complementary approaches. Diaminobenzidine (DAB) staining (Liu et al. 2014) of stem cross-sections from *Bdc3h/apx1* plants showed increased H_2_O_2_ accumulation compared with WT plants, as indicated by darker staining in the knockout lines (Figs. 5a and S7a). Overaccumulation of H_2_O_2_ was observed not only in lignified cell walls of interfascicular fibers and vascular bundles, but also in non-lignified parenchyma cells of the *Bdc3h/apx1* mutants. To further investigate the subcellular distribution of H_2_O_2_, we performed transmission electron microscopy (TEM) combined with cerium chloride (CeCl_3_) staining for in situ H_2_O_2_ localization (Czaninski et al. 1993; Fujita et al. 2020). This method enables the visualization of cerium perhydroxide precipitates formed upon reaction with H_2_O_2_. In *Bdc3h/apx1* plants, TEM images revealed higher deposition of H_2_O_2_ precipitates in the cell walls of sclerenchyma fibers (SF), XV, and parenchyma (P) cells, whereas H_2_O_2_ levels were lower in control plants (Fig. 5b). Notably, the precipitates were predominantly localized along the cytoplasmic side of the cell wall, particularly at the plasma membrane-cell wall interface (Fig. S8). Analysis of the TEM images confirmed significantly higher H_2_O_2_ accumulation in all cell-types in *Bdc3h/apx1* mutants (Fig. 5c). Furthermore, biochemical quantification using the Amplex Red assay confirmed that *Bdc3h/apx1* stems accumulate significantly higher H_2_O_2_ levels compared with WT plants (Figs. 5d and S7b). These findings suggest that the loss of BdC3H/APX disrupts redox homeostasis, leading to increased H_2_O_2_ accumulation and potential oxidative stress in these plants.

### Caffeate (CAF) rescues lignin and growth defects in *Bdc3h/apx1* mutants

**Figure 6:**
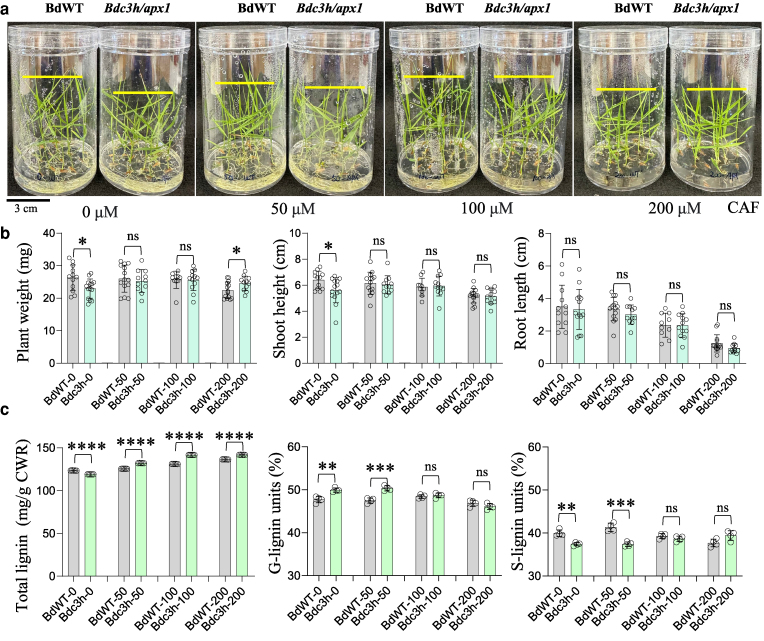
Effect of exogenous caffeic acid on growth and lignin phenotypes of *Bdc3h/apx1* plants. a) Growth phenotype of *BdWT* and *Bdc3h/apx1* plants grown in media supplemented with increasing concentrations of caffeate. b) Quantification of plant weight, shoot height, and root length of caffeic acid feeding plants (*n* > 10). c) Lignin content and composition of stem tissues following CAF treatment (*n* = 4). Error bars indicate mean ± Sd from biological replicates. Data points for all biological replicates are shown. Statistical analysis was performed using 1-way ANOVA followed by Dunnett's test for multiple comparisons: *P < 0.05; **P < 0.01; ***P < 0.001; ****P < 0.0001; ns, not significant.

The data presented above show that *BdC3H/APX* knockout plants display developmental defects together with reduced lignin content and elevated H_2_O_2_ levels. We next investigated whether exogenous CAF, the product of the C3H-catalyzed reaction, could rescue the mutant phenotypes. To test this, we conducted feeding experiments in which *Bdc3h/apx1* mutants were grown in media supplemented with increasing concentrations of CAF (0, 50, 100, and 200 *µ*m). CAF treatment significantly improved the growth of mutant plants in a dose-dependent manner. For example, at 50 and 100 *µ*m, *Bdc3h/apx1* plants showed increased shoot height and plant weight, bringing them closer to WT levels, whereas at 200 *µ*m, the *Bdc3h/apx1* mutants exhibited increased plant weight compared with WT plants (Fig. 6a and b). Additionally, when seedlings were grown on petri dishes, supplementation with 200 *µ*m CAF restored the shorter root phenotype of *Bdc3h/apx1* mutants to WT levels (Fig. S9a).

To further evaluate whether CAF feeding also restored the lignin phenotype, we analyzed the lignin content and composition of the same CAF-treated plants. In the absence of CAF, *Bdc3h/apx1* mutants exhibited lower lignin content and reduced S/G ratios compared with WT controls (Figs. 6c and S9b). However, CAF supplementation increased the total lignin content, particularly in mutants. For instance, at CAF concentrations of 50 and 100 *µ*m, the lignin content in the *Bdc3h/apx1* mutants surpassed that of WT plants (Fig. 6c). In addition to increasing lignin deposition, exogenous CAF also altered lignin composition in *Bdc3h/apx1* mutants. Specifically, CAF treatment led to a reduction in G-lignin and an increase in S-lignin subunits, resulting in an increasing trend of S/G ratio (Figs. 6c and S9b). At 100 and 200 *µ*m CAF, the proportion of G- and S-units, and S/G ratios in *Bdc3h/apx1* mutants were similar to those of WT plants (Figs. 6c and S9b). These results suggest that CAF supplementation compensates for the loss of *BdC3H/APX* function and restores the growth and lignin defects of the *Bdc3h/apx1* mutants.

### Catalase decreases H_2_O_2_ but fails to restore growth in *Bdc3h/apx1* mutants

We next asked whether elevated H_2_O_2_ levels alone could contribute to the growth defects observed in *Bdc3h/apx1* mutant plants. To test this, we performed similar feeding experiment with exogenous CAT supplementation. CAT can break down H_2_O_2_ into water and oxygen (Anjum et al. 2016; Ransy et al. 2020), thereby reducing H_2_O_2_ levels. WT and *Bdc3h/apx1* mutant plants were treated with different concentrations of CAT for 2 wk and their growth phenotypes were measured. Results show that treatment with a range of CAT concentration (0 to 200 units/mL) had no observable effect on the growth phenotype of *Bdc3h/apx1* mutant (Fig. S10a and b). The fresh weight and shoot height of the mutants were lower than those of the WT, and the growth phenotype was indistinguishable from that of untreated plants (Fig. S10a and b). Additionally, we measured H_2_O_2_ levels and found that exogenous CAT supplementation indeed reduced H_2_O_2_ levels in both genotypes (Fig. S10c). The decrease was more pronounced in *Bdc3h/apx1* mutants (∼30% reduction), yet the mutants still retained higher H_2_O_2_ levels compared with WT plants. These results show that although CAT treatment reduced H_2_O_2_ accumulation, it failed to rescue the growth defects in *Bdc3h/apx1* mutants, suggesting that their growth arrest is unlikely to be due to elevated H_2_O_2_ levels. While these experiments were designed to test whether reducing H_2_O_2_ levels could restore growth phenotype of *Bdc3h/apx1* mutants, exogenous CAT is unlikely to mimic the localization and functional role of BdC3H/APX in vivo.

**Figure 7:**
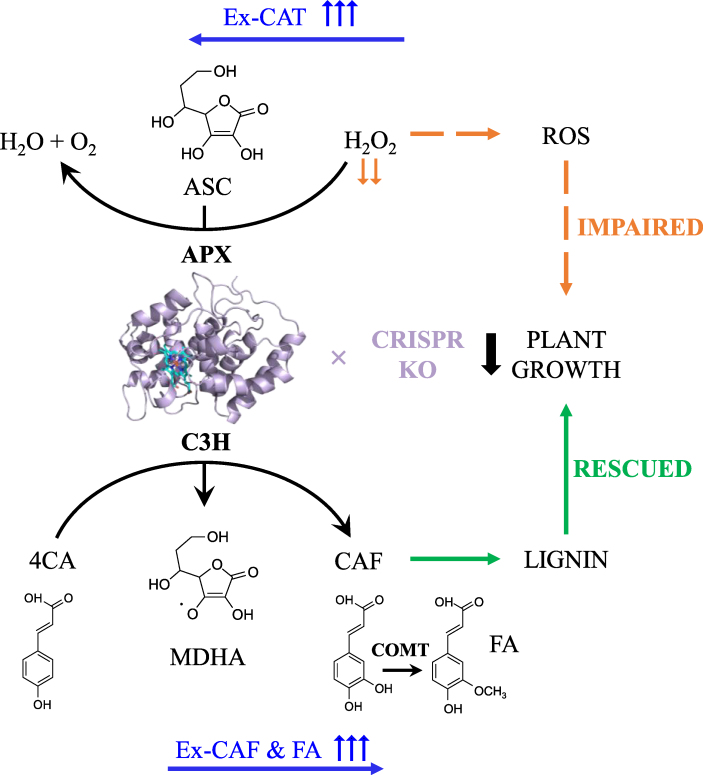
Proposed model of C3H/APX function in plant growth. Exogenous supplementation of CAT reduces H_2_O_2_ levels but fails to restore plant growth. In contrast, Ex-CAF, the direct product of the C3H reaction, restores lignin levels and rescues the growth phenotype, suggesting that impaired lignin biosynthesis is the primary cause of the developmental defects observed in C3H/APX-deficient plants ASC, ascorbate; MDHA, monodehydroascorbate; APX, ascorbate peroxidase, C3H, 4-coumarate 3-hydroxylase; COMT, caffeic acid *O*-methyltransferase; 4CA, 4-coumarate; CAF, caffeate; FA, ferulate.

It is possible that CAF act also as antioxidant and is more mobile than the CAT enzyme, and thus better able to rescue the *Bdc3h/apx1* mutant phenotypes. Therefore, to test whether the effect of CAF was due to its antioxidant activity, we repeated the feeding experiments with ferulic acid (FA), reported to have no significant antioxidant effect compared with CAF and other hydroxycinnamates (Chen and Ho, 1997). Similar to CAF treatment, supplementation with 50 *µ*m FA recovered the shoot height of the *Bdc3h/apx1* mutant to WT levels (Fig. S11a and b). Notably, the lignin content in the *Bdc3h/apx1* mutant was also restored at this concentration (Fig. S11c). At 100 mm FA concentration, both the total biomass and shoot height of the mutant were comparable to WT levels (Fig. S11a and b). Moreover, feeding of FA led to a reduction in the proportion of G-lignin and a consequent increase in the S/G ratio in the *Bdc3h/apx1* mutant (Fig. S11d). Statistical comparison within each genotype for both CAF and FA treatments showed that, in contrast to CAF, FA causes a severe growth inhibition, particularly at higher concentrations (100 and 200 *µ*m) in both WT and *Bdc3h/apx1* plants (Fig. S12a and b). Notably, this analysis highlights that the lignin levels of the *Bdc3h/apx1* mutants were notably increased at just 50 *µ*m FA or CAF (Fig. S12c). Altogether, the results from our feeding experiments show that exogenous CAF and FA supplementation restores lignin phenotype of BdC3H/APX1-deficient plants, suggesting that the developmental phenotypes observed in these mutants are, at least partially, due to impaired lignin biosynthesis (Fig. 7).

## Discussion

Prior studies have established CYP98A3 (*p*-coumaroyl shikimate/quinate 3′-hydroxylase) as the canonical C3′H in phenylpropanoid metabolism and lignin biosynthesis in *Arabidopsis* and *Populus*, where loss-of-function or down-regulation causes strong developmental and lignification phenotypes (Schoch et al. 2001; Franke et al. 2002a, 2002b; Coleman et al. 2008; Ralph et al. 2012). Analysis of *cyp98a3* insertion mutants in *Arabidopsis* revealed ectopic root lignification, despite complete loss of the canonical C3′H activity, providing genetic evidence for the existence of an alternative 3-hydroxylation route that may become active under stress conditions (Abdulrazzak et al. 2006). CYP98A3 is a membrane-associated cytochrome P450 that hydroxylates *p*-coumaroyl shikimate/quinate esters in the ER, whereas the C3H/APXs characterized in this study catalyze the ascorbate-dependent hydroxylation of free *p*-coumarate to caffeate in the cytosol. Because these enzymatic reactions differ in their substrates and subcellular localization, C3H/APX activity is not expected to compensate for the requirement for CYP98A3 during developmental lignification. Instead, we speculate that the cytosolic pathway involving C3H/APXs may operate in parallel to the canonical lignin pathway and contribute to detoxification of stress-induced ROS, connecting lignin biosynthesis with stress responses, plant defense, or acclimation pathways (Barros et al. 2019; Barros and Dixon, 2020).

While cytosolic APXs have well-established roles in ROS detoxification (Pnueli et al. 2003; Miller et al. 2007; Rosa et al. 2010; Guo et al. 2020), several plant developmental processes (Correa-Aragunde et al. 2013; Ribeiro et al. 2017; Mhamdi and Van Breusegem 2018; Huang et al. 2019), and responses to environmental stimuli (Caverzan et al. 2012; Pandey et al. 2017a), recent studies suggest that certain APX isoforms may also be involved in lignin biosynthesis (Barros et al. 2019; Zhang et al. 2022). Although this dual functionality has been supported using in vitro assays and gene knockdown studies (Barros et al. 2019; Zhang et al. 2022), genetic evidence from null mutants is currently lacking. In this study, we addressed this gap using CRISPR/Cas9 gene editing in *B. distachyon* and *P. tremula × P. alba* to functionally dissect the physiological role of cytosolic C3H/APX in vivo. Our results show that bifunctional C3H/APXs contribute to lignification, H_2_O_2_ detoxification, and normal plant development, and that simultaneous disruption of major C3H/APX genes is associated with severe growth defects in both monocot and dicot species. Further biochemical and genetic analyses support the conclusion that the developmental phenotypes typically observed in cytosolic APX-deficient plants (Pnueli et al. 2003; Rosa et al. 2010; Zhang et al. 2013; Carvalho et al. 2014; Guo et al. 2020), are primarily due to impaired lignin biosynthesis rather than redox imbalance.

Because *P. virgatum* and *S. bicolor* APXs (*Pavir.9KG480900* and *Sobic.001G410200,* Fig. S1a) have been previously characterized and reported to oxidize multiple phenylpropanoids with higher efficiency than the 3-hydroxylation reaction leading to formation of caffeate (Zhang et al. 2023a, 2023b), we revisited the C3H activity of BdC3H/APX1 to provide additional biochemical evidence supporting its C3H function (Barros et al. 2019). Our results show that the C3H activity in *B. distachyon* crude protein extracts is dependent on AsA and sensitive to buffer conditions. More specifically, C3H activity was lost in the presence of reducing agents such as DTT or BME, and when sodium phosphate buffer was replaced with Tris buffer (Fig. 1c). Tris buffer is a well-known ROS scavenger, often avoided in assays involving ROS biochemistry (Hicks and Gebicki 1986). A recent study has shown that Tris buffer can lead to false negatives by consuming ROS species before they can react with biological targets or analytical probes (Bansal et al. 2025). Similarly, thiol-based reducing agents such as DTT and BME are expected to suppress C3H/APX activity by quenching ROS and redox intermediates (Chen and Asada 1992). While our enzyme assays using crude protein extracts were highly active, the reactions using the recombinant BdC3H/APX protein required further optimization. The recombinant enzyme had to be reconstituted with hemin to show C3H activity (Fig. 1d). In contrast to prior studies using recombinant SbC3H/APX1 (*Sobic.001G410200*) and PvC3H/APX1 (*Pavir.9KG480900*), which reported weak caffeate production in the C3H reaction (Zhang et al. 2023a, 2023b) and used Tris-buffer (20 mm Tris, pH 7.5, 50 mm NaCl) for protein reconstitution, we maintained the enzyme in 50 mm sodium phosphate buffer (pH 6.0) without additional post-reconstitution purification steps, which may have helped preserve C3H activity. Although we included BSA in the assays for protein stability, we found it was not essential for maintaining C3H activity. Taken together, our biochemical data show that recombinant BdC3H/APX possesses bona fide C3H activity and that this activity requires AsA a hemin reconstituted version of the enzyme, and the absence of Tris buffer or strong reducing agents.

Despite extensive evidence that cytosolic APXs play key roles in redox regulation (Shigeoka et al. 2002; Davletova et al. 2005; Koussevitzky et al. 2008; Rosa et al. 2010; Yoshimura et al. 2024), their role in lignification remains poorly understood. To address this gap, we used CRISPR/Cas9 to generate null alleles in both monocot and dicot plant species. Prior research in *A. thaliana* relied on T-DNA insertional mutant lines (Pnueli et al. 2003; Suzuki et al. 2013; Barros et al. 2019), yet homozygous *AtC3H/APX1* mutants with insertions in coding regions could not be recovered (Barros et al. 2019). In *B. distachyon*, we previously identified activation-tagged lines with reduced expression of *BdC3H/APX1*, but T-DNA insertion mutants targeting the second homolog, *BdC3H/APX2*, were unavailable in the JGI mutant collection (Bragg et al. 2012; Barros et al. 2019). Similarly, in poplar, antisense constructs have been used to strongly suppress expression of mitochondrial APX (*PtomtAPX*), but complete knockout lines have not yet been reported (Zhang et al. 2022). A limitation of our genetic approach in *B. distachyon* was the inability to obtain single CRISPR/Cas9 knockout lines for *BdC3H/APX2*, likely due to the low efficiency of the second guide RNA used in our constructs. This restricted our analysis to *Bdc3h/apx1&2* mutant carrying monoallelic knockouts of *BdC3H/APX1* in combination with biallelic mutations in *BdC3H/APX2*. Future studies knocking out *BdC3H/APX2* alone will be necessary to better understand the functional redundancy and specific roles of C3H/APX family members.

A key finding of our study is that biallelic mutations in *C3H/APX1* in *B. distachyon* result in reduced lignin content, altered monolignol composition, and decreased plant growth. The milder growth phenotype of our CRISPR/Cas9- knockout lines compared with the previously reported T-DNA insertion lines (Barros et al. 2019) may reflect differences in growth conditions, as our plants were growth in controlled growth chambers whereas the TDNA lines were grown in greenhouse conditions, and ROS are particularly sensitive to light and temperature fluctuations (Choudhury et al. 2017; Fichman and Mittler 2020). These phenotypes were more pronounced in plants carrying a monoallelic mutation in *C3H/APX1* combined with biallelic mutation in *C3H/APX2* (*Bdc3h/apx1&2*), which exhibited a severe dwarf phenotype and failed to produce seeds (Fig. S4c and d). In *Populus*, the lignin and growth phenotypes of the monoallelic *C3H/APX1* mutants were indistinguishable from WT plants. However, biallelic *Ptc3h/apx1* mutants exhibited reduced plant height and a significant reduction in lignin content, particularly in syringyl (S) units. While we were able to generate calli with *Pt*C3H/APX1 and *Pt*C3H/APX2 double gene knockout in poplar, they failed to regenerate into shoots and eventually died in tissue culture (Fig. S6). Collectively, the inability to obtain viable double mutants in *Brachypodium* and poplar, the severe dwarfism observed in *Bdc3h/apx1&2* mutant, and previous reports of unsuccessful recovery of homozygous *Arabidopsis c3h/apx1* mutants carrying exon insertions (Barros et al. 2019), provide genetic evidence that complete loss of cytosolic C3H/APX activity could be lethal in plants. While the growth defects of the *Bdc3h/apx1* mutants were shown to be at least partially attributable to impaired lignin biosynthesis, we hypothesize that the lethality of the double *c3h/apx* mutants may also involve the PRX function of C3H/APX in ROS metabolism, as the mutants of upstream lignin biosynthetic pathway genes such as *PAL* or *C4H* exhibit severe dwarfism, but the plants remain viable and complete reproductive development (Schilmiller et al. 2009; Huang et al. 2010; Kim et al. 2021). Considering the insights gained from the crystal structures of cytosolic APX homologs in *P. virgatum* and *S. bicolor* (Zhang et al. 2023a, 2023b), it would be interesting to design a structure-guided mutagenesis approach to dissect the dual functionality of these bifunctional enzymes. Specifically, targeted substitutions at residues involved in either ascorbate or phenolic substrate binding, could help determine which catalytic activity is essential for plant development. Prime editing offers an ideal tool to introduce these point mutations in vivo (Molla et al. 2021; Gupta et al. 2024) and could be used to unambiguously test the dual catalytic function of C3H/APXs.

Consistent with the antioxidant role of APX enzymes and prior studies (Dunand et al. 2007; Zentgraf et al. 2022), we found significantly elevated H_2_O_2_ levels in the stems of *Bdc3h/apx1* mutants. At 60 DAP, mutants accumulated ∼20 *µ*mol H_2_O_2_/mg tissue, nearly 3-fold higher than WT plants (∼7 *µ*mol/mg). Although H_2_O_2_ levels declined by 90 DAP, concentrations in mutant stems (∼3.5 *µ*mol/mg) remained slightly higher than in the controls (∼3.0 *µ*mol/mg). These findings show that loss of C3H/APX function leads to H_2_O_2_ overaccumulation, but that the magnitude of this difference declines as plants transition from vegetative to reproductive growth. While leaf senescence is typically associated with increased ROS levels (Guo and Gan 2012; Rogers and Munné-Bosch 2016; Mhamdi and Van Breusegem 2018), this pattern may differ in stem tissues. One possible explanation is a developmental increase in CAT or glutathione reductase activity during stem maturation, which has been shown to enhance H_2_O_2_ catabolism (Queval and Noctor 2007; Costa et al. 2010). Our ultrastructural analysis using TEM further revealed localized H_2_O_2_ precipitates mainly along the cytoplasmic side of the cell wall, adjacent to the plasma membrane in stem vascular cells (Fig. S8). This distribution aligns with the membrane localization of NADPH oxidases (RBOHs), which generate superoxide $\left(O_{2}^{-}\right)$ that is converted into H_2_O_2_ by superoxide dismutases (SODs) (Yoshioka et al. 2003; Kadota et al. 2014; Mittler et al. 2022). Of the many classes of plant SODs, some are localized to the apoplast and have been proposed to be involved in generation of H_2_O_2_ for lignification (Ogawa et al. 1996; Kasai et al. 2006). The absence of H_2_O_2_ in the apoplastic side of the membrane may reflect rapid scavenging by cell-wall PRXs, which utilize H_2_O_2_ for oxidative polymerization during lignin biosynthesis (Dixon and Barros 2019). A better understanding of how plants regulate this H_2_O_2_ production and scavenging near the plasma membrane may provide insights into how plants regulate lignification under both developmental and stress-induced conditions.

While immunogold labeling revealed that cytosolic APXs are mainly localized in the cytosol with only minimal labeling detected in the apoplast of mature soybean nodule cells (Dalton et al. 1993), their subcellular localization could be developmentally regulated. Future work should address whether C3H/APXs undergo subcellular relocalization in vascular tissues, similar to mitochondrial PtomtAPX, which has been shown to translocate to the apoplast during early lignification stages (Zhang et al. 2022). In contrast to PtomtAPX, we observed consistently reduced lignin and H_2_O_2_ levels in C3H/APX-deficient plants at both early vegetative (60 DAP) and reproductive stages (90 DAP), suggesting that the role of cytosolic C3H/APXs is not compensated by Class III PRXs or LACs during later developmental stages. Moreover, given their capacity to oxidize monolignol intermediates (Zhang et al. 2022, 2023a, 2023b), cytosolic APXs must be compartmentalized to prevent the ectopic oxidation of phenolics in the cytosol. It is possible that such compartmentalization could involve vesicle-mediated trafficking, as proposed for the transport of PtomtAPX from mitochondria to the cell wall during programmed cell death (Zhang et al. 2022; Kankaanpää et al. 2024). Interestingly, recent proteomic and metabolomic analyses of extracellular vesicles from lignified Norway spruce (*Picea abies*) cells identified AsA-dependent oxidases together with lignin monomers (Kankaanpää et al. 2024). Consistent with this model, the pH values within secretory and endosomal vesicles are lower than in the cytosol, typically in the pH 5.5 to 6.5 range (Shen et al. 2013), which are compatible with the buffer conditions used in our biochemical assays and previously reported C3H activities in plants (Vaughan and Butt 1970; Duke and Vaughn 1982; Kojima and Takeuchi, 1989).

In conclusion, our results support a model in which cytosolic C3H/APXs function in both ROS detoxification and phenylpropanoid metabolism, linking these roles to plant growth and development (Fig. 7). Using CRISPR/Cas9-mediated knockouts in model monocot and woody dicot systems we reveal that loss of C3H/APX1 impairs growth and lignification, and complete loss of C3H/APX activity could be lethal in plants. In addition, we show that *B. distachyon* C3H/APX1 catalyzes the ascorbate-dependent 3-hydroxylation of 4-coumarate and found that H_2_O_2_ accumulation occurs specifically in the cytoplasmic side of the cell wall, adjacent to the plasma membrane of vascular cells. Although exogenous CAT decreased H_2_O_2_ levels, it failed to rescue growth, indicating that antioxidant activity alone is insufficient to compensate for C3H/APX function. In contrast, caffeate supplementation restored both lignin biosynthesis and plant growth, supporting the notion that impaired lignin biosynthesis is a key driver of the developmental phenotype observed (Fig. 7). Altogether, these findings advance our understanding of the molecular cross-talk between lignin and redox metabolism during plant development, offering a potential strategy for engineering biomass yield and stress resilience in bioenergy and forage crops.

## Materials and methods

### Phylogenetic analysis

Homologous C3H/APX genes were identified from publicly available genome databases for *B. distachyon*, *Medicago truncatula*, *Oryza sativa* (rice), *Zea mays* (maize), *S. bicolor*, and *P. trichocarpa*. Protein sequences with >70% amino acid identity to *A. thaliana* C3H/APX1 (At1g07890) were selected for further analysis. Multiple sequence alignments were performed using CLUSTALX v1.81 (Thompson et al. 1997) and refined with BIOEDIT v7.0.5 (Hall 1999). Phylogenetic tree was constructed using the neighbor-joining method implemented in MEGA v5.0 (Tamura et al. 2011), with bootstrap analysis conducted using 1,000 replicates to assess branch support.

### Crude protein extracts and enzyme activity assays

The crude protein extracts and reaction mixtures used to assay C3H activity were prepared using previously described protocols (Kojima and Takeuchi, 1989; Barros et al. 2019). Briefly, crude ammonium sulfate extracts were prepared by grinding in liquid nitrogen and homogenizing 2 g of fresh plant tissue in 6 mL of 50 mm sodium phosphate buffer (pH = 6) in the presence of 1 g of polyvinylpolypyrrolidone (PVPP) and 1 g of 0.5 mm acid-washed glass beads. The samples were incubated overnight on a tube rotator at 4 °C. After filtration through 4 layers of Mira cloth (Millipore) and centrifugation at 500*×g* for 1 min to remove the PVPP, glass beads, and cell debris, ammonium sulfate was added to a final concentration of 0.5 g/mL. The resulting pellet was resuspended in half the original volume in 50 mm phosphate buffer (pH = 6.0). The homogenates were assayed directly. The reaction mixture with the crude protein extract contains 10 *μ*L of 10 mm 4-coumarate (4CA), 7.5 *μ*L of 10 mg/mL BSA, 20 *μ*L of 20 mM l-ascorbate, 7.5 *μ*L of 1 M sodium phosphate buffer (pH 6.0), 15 *μ*L of the protein crude *Brachypodium* stem extracts (0.7 *μ*g/*μ*L) and water up to a total volume of 100 *μ*L. For the in vitro assays with the recombinant enzyme, the basic reaction mixture contains 10 *μ*L of 10 mm 4-coumarate, 7.5 *μ*L of 10 mg/mL BSA, 20 *μ*L of 20 mM l-ascorbate, 7.5 *μ*L of 1 M sodium phosphate buffer (pH 6.0), 10 *μ*L of 0.3% H_2_O_2_ solution, 500 ng of the hemin-reconstituted (as described below) purified recombinant *Brachypodium* C3H and water up to a total volume of 100 *μ*L. The mixture was incubated for 2 h at 30 °C while shaking and stopped by adding 10 *µ*L of acetic acid. Incubating the reaction at a pH higher than 8, or preparing the crude protein extracts with antioxidants such as DTT or 2-mercaptoethanol, or the stock solutions with dimethylsulfoxide (DMSO), completely inhibited C3H activity. Stock solutions were prepared in 50 mm phosphate buffer (pH = 6), and 4-coumarate was dissolved by sonication and heated (10 min at 96 °C) with shaking.

### Recombinant expression of C3H/APX

The full-length cDNA sequences for *Bradi1g65820* (*BdC3H/APX1*) were obtained from Phytozome v12.0 and amplified by RT-PCR (Phusion HiFi polymerase; New England BioLabs) from stem tissue cDNA of WT *Brachypodium* plants. Total RNA was extracted using Trizol reagent (Invitrogen), and first-strand cDNA was synthesized using the SuperScript III First-Strand System for RT-PCR (Invitrogen) following the manufacturer's protocol. The cDNAs were cloned into the pENTR-D Topo vector and subsequently into the pDEST17 vector via LR recombination, resulting in a 6xHis-C3H fusion construct. The 6xHis-tagged C3H protein was expressed in *E. coli* strain Rosetta, grown in Luria-Bertani (LB) medium containing 0.1 mg/mL carbenicillin at 37 °C. When the optical density at 600 nm reached 0.7 to 0.9, protein expression was induced using 0.5 mm IPTG, and cells were grown at 16 °C for 18 h. The cells from a 25 mL culture were harvested by centrifugation and resuspended in 2 mL extraction buffer (50 mm Tris-HCl, pH = 8.0, 500 mm NaCl, and 10 mm imidazole). All subsequent steps were performed at 4 °C. Cell lysis was carried out using an ultrasonic homogenizer (Model-120, Fisher Scientific), and the lysates were clarified by centrifugation at 16,000*×g* for 20 min. Ni-NTA beads (Qiagen) were added, and the mixture was incubated at 4 °C for 30 min with constant inversion. After washing the beads 3 times with extraction-washing buffer, the target proteins were eluted with 250 *µ*L of elution solution (50 mm Tris-HCl, pH = 8.0, 500 mm NaCl, 250 mm imidazole). Protein purity was verified by SDS-PAGE.

Reconstituted C3H was prepared following a modified protocol originally developed for soybean cytosolic APX (Dalton et al. 1996; Barros et al. 2019). Briefly, purified BdC3H/APXs were exchanged in 50 mm sodium phosphate buffer (pH = 6) and concentrated using a 10 kDa Amicon concentrator. Bovine hemin (10 mg, Sigma: CAS 16009-13-5) was dissolved in 1 mL of 10 mm NaOH and diluted to 10 mL with phosphate buffer. The hemin solution (1 mL) was gradually added dropwise to 4 mL of C3H solution with gentle stirring. After 15 min of incubation at 4 °C, the solution was centrifuged at 12,000*×g* for 10 min to remove any denatured products. The concentration of the reconstituted holoenzymes in the supernatant was determined using the Bradford assay and directly used for enzymatic assays as described above.

### CRISPR/Cas9 gene editing in *Brachypodium*

sgRNAs were designed using CRISPR-P software (Lei et al. 2014) to target 2 *C3H/APX* genes: *BdC3H/APX1* (*Bradi1G65820*) and *BdC3H/APX2* (*Bradi1G16510*). The spacer sequence of sgRNA1 for *BdC3H/APX1* was cloned at *Btg*ZI restriction site downstream of OsU6.1 promoter and the spacer sequence of sgRNA2 for *BdC3H/APX2* was cloned at *Bsa*I restriction site downstream of OsU6.2 in pENTR-gRNA1 vector based on the protocol as described (Char et al. 2019). The resulting 2 sgRNA expression cassettes were recombined via LR clonase into the binary destination vector pJD633-GW, a plasmid with addition of a Gateway recipient cassette to JD633. JD633 harbors the Cas9 gene under *ZmUbi* promoter and the *TaGRF1-GIF* chimera to enhance regeneration efficiency during plant transformation (Debernardi et al. 2020). The final construct, designated pJD633-EV1 (C1), was verified by PCR using sgRNAs and Cas9-specific primers (Table S3) and confirmed by Sanger sequencing prior to transfer into *Agrobacterium tumefaciens* strain EHA105.

Plant transformation was performed following previously described protocols with some modifications (Vogel and Hill 2008; Alves et al. 2009). Briefly, surface-sterilized mature seeds from ∼100-d-old *B. distachyon* (Bd21-3) plants were plated on callus induction medium (CIM, Murashige and Skoog Basal Medium with Vitamins, 4.43 g/L; sucrose, 30 g/L; 2,4-Dichlorophenoxyacetic acid, 2.5 mg/L; agar, 8 g/L; pH 5.8) in round petri plates and incubated at 28 °C in darkness for 4 wk. Emerging calluses were excised, fragmented and transferred to new CIM for an additional 2 wk under the same conditions. Vitreous and powdery parenchymatous cell masses were removed during subculturing. Embryogenic calli were subdivided and maintained in CIM for one more week prior to transformation. The suspension culture of *A. tumefaciens* (strain EHA105) was applied directly onto the calli, and the mixture was gently agitated on a rotary shaker for 30 to 45 min. Afterward, excess liquid was removed with sterilized filter paper. The inoculated calli were then transferred to co-cultivation medium (CIM supplemented with acetosyringone at 100 *μ*m), sealed with Parafilm, and incubated in darkness at 26 °C for 2 d to promote *Agrobacterium*-mediated gene transfer. Following the 2-d co-cultivation period, calli were transferred to selection medium containing hygromycin (CIM supplemented with timentin at 300 mg/L, and hygromycin at 50 mg/L). Hygromycin-resistant calli were moved to the first regeneration medium (MS Basal Medium with Vitamins, 4.43 g/L; sorbitol, 50 g/L; 6-benzylaminopurine 3 mg/L; 1-naphthaleneacetic acid, 0.5 mg/L; hygromycin, 50 mg/L; agar, 16 g/L; pH 5.8) and incubated at 25 °C in complete darkness for 4 to 5 wk. The proliferating calli were then transferred to a second regeneration medium (MS Basal Medium with Vitamins, 4.43 g/L; sucrose, 30 g/L; 6-benzylaminopurine, 3 mg/L; 1-naphthaleneacetic acid, 0.5 mg/L; timentin 150 mg/L; hygromycin, 25 mg/L; Agar, 8 g/L; pH 5.8) and cultured for 2 to 3 wk. Calli were subcultured every 2 to 3 wk until shoots regeneration occurred. Developing shoots were transferred to rooting medium (MS Basal Medium with Vitamins, 4.43 g/L; sucrose, 30 g/L; hygromycin, 25 mg/L; Agar 8 g/L; pH 5.8) and maintained under a 16-h light/8-h dark photoperiod at 25 °C for 3 wk. Plantlets reaching 1 to 2 cm with visible roots were moved to plastic cups containing fresh rooting medium to allow further development. Once plantlets reached 3 to 4 cm height, the cups were gradually opened, and distilled water was added to acclimate the plants at room temperature for 1 to 2 d. Fully rooted plantlets were transplanted into soil-filled pots and grown in a greenhouse at 28 °C under a 16-h light photoperiod. Leaf tissue was harvested at this stage for genomic DNA extraction using the CTAB method and PCR-based genotyping.

To identify regenerated T_0_ plantlets harboring CRISPR/Cas9 construct (C1) targeting *BdC3H/APX1* and *BdC3H/APX2*, we used a dual-target PCR screening strategy. A single amplification was performed using gC3H/APX1-F1 and gC3H/APX2-R1 primers (Table S3). Positive transformants were identified by comparing DNA band patterns to those of the corresponding Gateway clones (positive control) and WT *B. distachyon* DNA (negative control). To assess genome editing at the target loci, PCR amplification was performed using gene-specific primers (Table S3). For rapid detection of indels in the T_0_ generation, we used a PCR-Restriction enzyme assay (Ashrafi et al. 2018), selecting restriction sites overlapping predicted CRISPR/Cas9 cleavage sites nearby PAM sequences. Edited alleles were verified through sequencing. In subsequent T_1_ and T_2_ generations, gene editing events were confirmed via Sanger sequencing.

Mature seeds used for transformation and callus induction were collected from plants grown in a greenhouse for ∼100 d under controlled conditions (28 °C, 16 h light/8 h dark photoperiod). The plants were grown in 12-inch pots with Pro-Mix soil. NPK fertilizer with a composition of 15-6-9 was applied as needed. Regenerated transgenic lines were transferred to a growth chamber and maintained under the same photoperiod (16-h light/8-h dark) at 22 to 25 °C and light conditions at 200 to 250 *µ*mol m^−2^ s^−1^. Homozygous T_2_ generation *Bdc3h/apx1* mutant lines were used for phenotypic and biochemical analysis. A total of 57 independent T_0_ lines were screened to obtain the *Bdc3h/apx1&2* mutant. Because this genotype was infertile, only a single T_1_ plant was recovered and used for phenotypic characterization.

### CRISPR/Cas9 gene editing in *Populus*

Two constructs were prepared to edit *PtaC3H/APX1* alone or *PtaC3H/APX1* and *PtaC3H/APX2* simultaneously in hybrid poplar. Gene-specific gRNAs targeting variant-free regions of the hybrid genome (*PtaC3H/APX1*: GCAAAAACCGGTGGGCCCTTCGG and *PtaC3H/APX2*: ACCAAGACAGGAGGGCCATTTGG) were vetted against the *P. tremula × alba* INRA 717-1B4 genome (Zhou et al. 2023) using the *Populus* VariantDB (Zhou et al. 2025). The *PtaC3H/APX1* gRNA oligonucleotide pair with vector homology (Table S3) was cloned into the p201N-Cas9 binary vector (Addgene no. 59175) under the control of the *M. truncatula* U6.6 (MtU6.6) promoter using NEBuilder HiFi DNA Assembly Cloning Kit (New England Biolabs) as described in Bewg et al. (2022). For double knockout, a synthetic fragment (Twist Bioscience, Table S3) was assembled into pORE303N (Addgene no. 194438) (Ortega et al. 2022), with the *PtaC3H/APX1* gRNA driven by MtU6.6 and the *PtaC3H/PAX2* gRNA driven by the *A. thaliana* AtU3d promoter. *A. tumefaciens*-mediated transformation of *Populus* 717 leaf explants and subsequent plant regeneration were performed according to established protocols (Bewg et al. 2022).

Genomic DNA was extracted from leaves or calli of independent transgenic events, and amplicon sequencing libraries were prepared as described (Bewg et al. 2022) using consensus primers (Table S3). Amplicons were barcoded using Illumina indexing primers, pooled, and sequenced on an Illumina MiSeq platform (PE150) at the Georgia Genomics and Bioinformatics Core Facility, University of Georgia (RRID:SCR_010994). Sequencing reads were processed using the AGEseq (Analysis of Genome Editing by Sequencing) pipeline (Xue and Tsai 2015), with the mismatch tolerance set to 1%, followed manual curation.

Multiple independent *Ptc3h/apx1* lines, along with WT and Cas9 vector control lines, were propagated from tissue culture plants. Plants were grown in 1-gallon pots containing a commercial soil mix (Fafard 3B) supplemented with slow-release fertilizer (Osmocote 15-9-12 NPK). For lignin phenotyping, plants were grown in a greenhouse until they reached ∼1.5 m in height. Irrigation was applied every other day, and supplemental LED lighting was used to maintain a 16-h photoperiod with a minimum photosynthetic photon flux density of ∼600 *µ*mol m^−2^ s^−1^. Growth phenotype images were taken at an earlier stage (< 30 cm tall, Fig. 4a) to capture the initial developmental differences between mutant and control lines.

### Microscopy and histochemical staining

Light microscopy of poplar xylem cells was performed using the EVOS XL Core Imaging System (Thermo Fisher Scientific). Lignin autofluorescence was visualized under UV excitation using an EVOS FL Cell Imaging System equipped with a DAPI LED light cube (excitation: 357/44 nm; emission: 447/60 nm). For histochemical analysis, mature stem segments (2 cm basal regions) from WT and mutant *B. distachyon* plants were sectioned into 70 *μ*m-thick slices using a Leica VT100S vibrating blade microtome. Phloroglucinol–HCl (Wiesner) staining was conducted with a 2% (w/v) phloroglucinol solution in ethanol, mixed with 12 N HCl in a 2:1 ratio (Pradhan Mitra et al. 2014). H_2_O_2_ was detected by DAB (3,3′-Diaminobenzidine) staining method (Thordal-Christensen et al. 1997). Fresh basal stem segments were sliced into 70 *μ*m-thick sections with a Leica vibratome (VT1000S) and immediately incubated in 1 mg/mL DAB solution (pH = 3.8) for 2 h. Samples were then destained in 100% ethanol for 10 min. H_2_O_2_ was detected as a reddish-brown precipitate under light microscopy. Brightfield and UV fluorescence images were acquired using the Echo Revolve R4 microscope (Discover Echo).

### Lignin analyses

Lignin composition (H%, G%, S%, and S/G ratio) was determined using the thioacidolysis method, following stablished protocols (Barros et al. 2019; Chen et al. 2021). Briefly, cell wall residues (CWRs) samples were prepared by sequential extractions using methanol, chloroform/methanol (2:1) and MQ water. Samples were then freeze-dried overnight to remove residual moisture. For thioacidolysis, 2.90 to 3.10 mg of each CWR sample was incubated with 2 mL of freshly prepared thioacidolysis reagent, consisting of dioxane:ethanethiol:boron trifluoride etherate in an 87.5:10:2.5 ratio. Reactions were carried out for 3 h at 103 °C in a heating block. Following incubation, 2.7 mL of MQ water, 0.57 mL of saturated NaHCO_3_ solution, 20 *µ*L of 0.3% (w/v) docosane (internal standard), and 2 mL of chloromethane were sequentially added to each sample. Reaction mixtures were transferred to clean glass vials and evaporated under a stream of nitrogen gas. To derivatize the lignin monomers, 100 *µ*L of a 1:1 mixture of pyridine and BSTFA containing 1% TMCS was added to each vial. After derivatization, 10% of the supernatant was used for lignin composition analysis. Lignin-derived monomers were identified and quantified by GC–MS (Agilent 8890 GC system) using characteristic mass-to-charge ratios: 239 m/z for *p*-hydroxyphenyl (H) units, 269 m/z for guaiacyl (G) units, and 299 m/z for syringyl (S) units. The internal standard docosane was monitored at 57 m/z.

Total lignin content was determined using the acetyl bromide method (Moreira-Vilar et al. 2014). Approximately ∼5 mg of the lyophilized CWR above was weighed into screw-cap centrifuge tubes and incubated with 0.5 mL of 25% (v/v) acetyl bromide in glacial acetic acid at 70 °C for 30 min. Samples were then chilled on ice for 10 min, and a 20 *µ*L aliquot of the supernatant was transferred to a quartz cuvette and mixed with 380 *µ*L of a stopping reagent composed of 2 M NaOH, 5 M hydroxylamine-HCl, and glacial acetic acid in a ratio of 0.9:0.1:6 (v/v/v). Absorbance was measured at 280 nm using a Genesys 10S UV-Vis spectrophotometer (Thermo Fisher Scientific). Lignin concentration was quantified based on a standard curve generated from known amounts of an alkali lignin (Sigma, CAS 8068-05-1) standard processed in parallel.

### Detection of H_2_O_2_ with the Amplex red peroxide assay

Hydrogen peroxide (H_2_O_2_) levels in stem, root, and leaf tissues were quantified using the Amplex Red Hydrogen Peroxide/Peroxidase Assay Kit (Invitrogen, Thermo Fisher Scientific) following the manufacturer's protocol. Fresh tissue samples were flash-frozen in liquid nitrogen and thoroughly ground to a fine powder. Each sample was homogenized in 200 *µ*L of 0.1% (w/v) trichloroacetic acid and centrifuged, and 50 *µ*L of the supernatant was transferred to a black 96-well microplate. A standard curve was generated by diluting 20 mm H_2_O_2_ stock solution in 1× reaction buffer to obtain final concentrations ranging from 0 to 10 *µ*m (50 *µ*L per well). A 5 mL working solution was freshly prepared by combining 50 *µ*L of 10 mm Amplex Red reagent stock, 100 *µ*L of 10 U/mL horseradish peroxidase (HRP), and 4.85 mL of 1× reaction buffer. Subsequently, 50 *µ*L of the Amplex Red/HRP working solution was added to each well containing standards, controls, and samples. Plates were incubated for 30 min at room temperature in the dark. Fluorescence was measured using a Qubit 4 fluorometer (Invitrogen, Thermo Fisher Scientific). After the assay, samples were dried in a 50 °C oven for 24 h and weighed. H_2_O_2_ levels were normalized to the dry weight of the tissue and expressed as nmol H_2_O_2_ per mg of dry tissue.

### TEM visualization of H_2_O_2_ deposits by the cerium chloride assay

Hydrogen peroxide accumulation in *B. distachyon* stem tissues was detected following described protocols (Czaninski et al. 1993; Fujita et al. 2020), with minor modifications. Fresh stem sections (70 *µ*m) were collected from WT and *Bdc3h/apx1* knockout plant and processed for TEM as follows. Cross-sections from the basal 1 cm of the main stems were incubated with 50 mm 3-[N-morpholino] propane sulfonic acid (Mops; pH = 7.2) containing freshly prepared 10 mm cerium chloride (CeCl_3_) for 30 min. Sections were washed twice in MOPS buffer for 5 min and fixed in glutaraldehyde solution 2.5% in 100 mm phosphate buffer (pH = 7.4) for 1 h at room temperature and subsequently fixed in 2% paraformaldehyde, 2% glutaraldehyde in 1 × phosphate buffered saline, pH = 7.35 (Fisher Scientific). Next, fixed tissues were rinsed with 1 × PBS, pH = 7.35. Secondary fixation was performed using 1% osmium tetroxide (Ted Pella, Inc., Redding, CA, USA) with 1.5% potassium ferrocyanide in PBS. The specimens were next incubated at room temperature for 1 h, protected from light. Then rinsed with cacodylate buffer and further with distilled water. A graded dehydration series was performed using ethanol (40 min each at 30%, 50%, and 70%), then 100% ethanol twice for 1 h each. Dehydrated tissues were then infiltrated with 1/3 then 2/3 Spurrs resin/ethanol solutions overnight, then for 4 h respectively. Next, samples were incubated in 3 exchanges of pure Spurrs resin totaling 24 h and polymerized at 60 °C for 48 h. Sections were cut to a thickness of 50 nm using an ultramicrotome (Ultracut UCT, Leica Microsystems, Germany) and a diamond knife (Diatome, Hatfield, PA, USA). Images were acquired with a JEOL JEM 1400 TEM (JEOL, Peabody, MA, USA) at 80 kV on a Gatan RIO 9 camera (Gatan, Inc., Pleasanton, CA, USA). Reagents were purchased from Electron Microscopy Sciences and specimen preparation was performed at the Electron Microscopy Core Facility at the University of Missouri.

### Exogenous feeding experiments

A 20 mm caffeic acid stock solution was prepared by dissolving 10.8 mg of caffeic acid in 250 *μ*L DMSO, followed by addition of 2.75 mL ultrapure MQ water. The solution was then filter-sterilized. A control solution was prepared with 3 mL of MQ water containing 250 *μ*L DMSO. The caffeic acid solution was further diluted to the target experimental concentration using the control solution. Similarly, FA working solutions were prepared as described for caffeic acid. For the CAT experiments, 4,000 units/mL (1 mg/mL) stock solution was made by dissolving CAT from bovine liver (Sigma, CAS 9001-05-2) in MQ water, followed by filtration. The solution was then diluted to the desired concentration as needed for the experiment. The lemma and palea were peeled off prior to sterilization of *B. distachyon* (Bd21-3) seeds with 75% ethanol for 5 min, and rinsed 3 times with sterile water, treated with 15% bleach for 5 min, and rinsed again 3 times with sterile water. MS medium containing different concentrations of caffeic acid, FA, or CAT was prepared, and the surface-sterilized seeds were placed flat on the medium and kept in the dark at 4 °C for 2 d before being transferred to a plant growth chamber. Lignin and plant growth phenotypic analyses were conducted after 2 wk. For each treatment and genotype, 3 independent culture tubes were prepared. All plants were used for plant weight, shoot height, and root length measurements. For lignin analyses, stems from all plants were pooled and analyzed by thioacidolysis followed by GC-MS in 4 independent biological replicates.

### Statistical analysis

Statistical tests were performed using GraphPad Prism 10 (version 10.4). *P*-values were calculated by unpaired 2-tailed Student's *t*-test between 2 groups or by 1-way analysis of variance (ANOVA) when comparing 2 groups among 3 or more groups. Individual data points for all biological replicates are shown in the figures. Statistical significance is denoted as follows: *P < 0.05, **P < 0.01, ***P < 0.001, and ****P < 0.0001.

### Accession numbers

Sequence data from this article can be found in Phytozome/JGI and the GenBank/EMBL data libraries under the following gene IDs and accession numbers: BdC3H/APX1 (Bradi1g65820; XM_003558130); BdC3H/APX2 (Bradi1g16510; XM_010235217); PtC3H/APX1 (Potri.009G015400; XM_052455780); PtC3H/APX2 (Potri.016G084800; XM_002322815); PtC3H/APX3 (Potri.006G132200; XM_006381438).

## Supplementary Material

kiag293_Supplementary_Data

## Data Availability

The data underlying this article are available in the article and in its online supplementary material.
