# Supplementary material for: Conserved 4-coumarate 3-hydroxylase/ascorbate peroxidase bifunctionality coordinates lignin deposition and plant growth in Brachypodium and Populus
Source: Plant Physiol. 2026 May 22;201(2):kiag293. doi: 10.1093/plphys/kiag293 (PMC13273577; doi:10.1093/plphys/kiag293)
Supplement: kiag293_Supplementary_Data [file kiag293_Supplementary_Data.pdf]

Supplementary Data

Conserved C3H/APX bifunctionality coordinates lignin deposition and plant growth in *Brachypodium* and *Populus*

Weiwei Zhu[1], Harmeet Singh-Bakala[1], Bo Liu[1], William Bewg[2,3], Max Bentelspacher[1], Rachel A. Weber[1], María Ángeles Peláez-Vico[1,4], Margot S.S. Chen[2,3], Chung-Jui Tsai[2,3,5,6], Ron Mittler[1,4], Bing Yang[1,7], and Jaime Barros[1*]

*Corresponding author. Email: jaime.barros@missouri.edu

Supporting figures

Supplementary Figure S1. Diversification of C3H/APXs and CRISPR-Cas9 target genes in this project.
Supplementary Figure S2. C3H/APX catalyzes the enzymatic hydroxylation of 4-coumarate (4CA) to caffeate (CAF) using ascorbate (AsA) as electron donor.
Supplementary Figure S3. Sequencing results of *Brachypodium distachyon BdC3H/APXs* gene edited lines.
Supplementary Figure S4. Plant growth phenotype of *Bdc3h/apx* plants.
Supplementary Figure S5. Lignin phenotype of *Bdc3h/apx1* plants at 90 days after planting.
Supplementary Figure S6. Plant phenotype of the *Ptc3h/apx* plants.
Supplementary Figure S7. H_2O_2 accumulation in different tissues of Brachypodium wild-type (BdWT) and *Bdc3h/apx1* mutants at 90 days after planting.
Supplementary Figure S8. Subcellular localization of H_2O_2 in Brachypodium wild-type stem tissues.
Supplementary Figure S9. Effect of exogenous caffeic acid on growth and lignification of *Bdc3h/apx1* plants.
Supplementary Figure S10. Phenotypes of *Bdc3h/apx1* plants feed with catalase.
Supplementary Figure S11. Effect of exogenous ferulic acid on growth and lignification of *Bdc3h/apx1* plants.
Supplementary Figure S12. Effects of caffeic acid and ferulic acid on plant growth and lignin content in wild type and *Bdc3h/apx1* plants.

Supporting tables

Supplementary Table S1. CRISPR/Cas9 mutation patterns of *PtC3H/APX1* alleles in poplar lines.
Supplementary Table S2. CRISPR/Cas9 mutation patterns of *PtC3H/APX1&2* alleles in poplar callus lines.
Supplementary Table S3. Sequences of the primers used in this work.

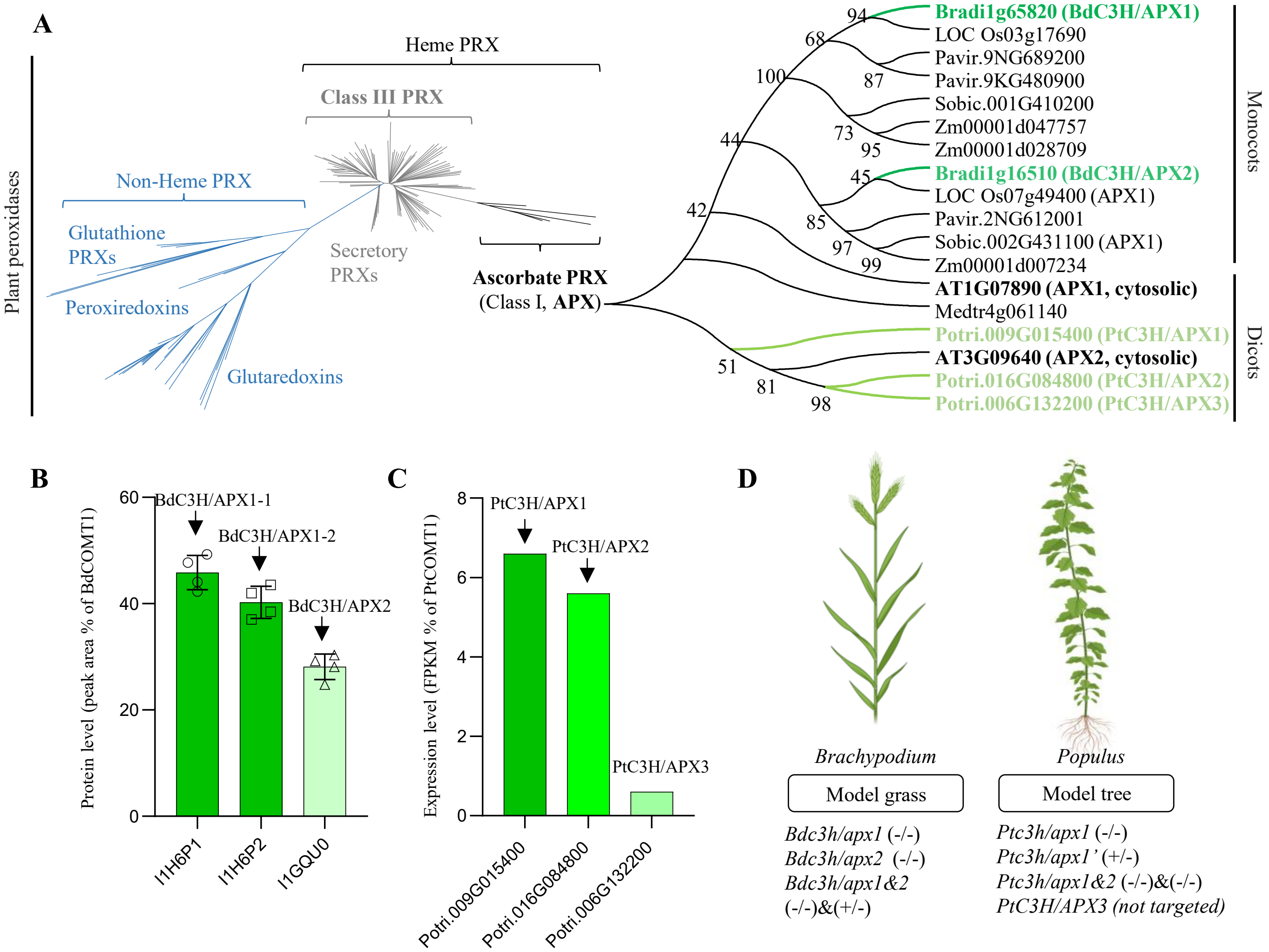

Supplementary Figure S1. Diversification of C3H/APXs and CRISPR-Cas9 target genes in this project. (**A**) Phylogenetic clustering of plant peroxidases (left) and phylogenetic tree of Class I cytosolic ascorbate peroxidases (APXs) proteins from monocot and dicot species (right), including *Zea mays* (Zm), *Sorghum bicolor* (Sobic), *Brachypodium distachyon* (Bradi), *Oryza sativa* (Os), *Arabidopsis thaliana* (AT), *Medicago truncatula* (Medtr), *Phaseolus vulgaris* (Pavir) and *Populus trichocarpa* (Potri). Proteins characterized in this study are highlighted in green. (**B**) Protein expression level of BdC3H/APXs in the stems of *Brachypodium* using the highly abundant lignin protein COMT1 as reference protein. Error bars indicate mean ± SD. (**C**) Gene expression levels of *PtC3H/APXs* in the xylem of *Populus* (FPKM, fragments per kilobase of transcript per million mapped reads), using the expression levels of poplar *COMT1* as reference gene. (**D**) CRISPR-Cas9 targeted editing of C3H/APXs in *Brachypodium distachyon* and *Populus tremula × alba* INRA 717-1B4. (-/-) homozygous; (+/-) heterozygous.

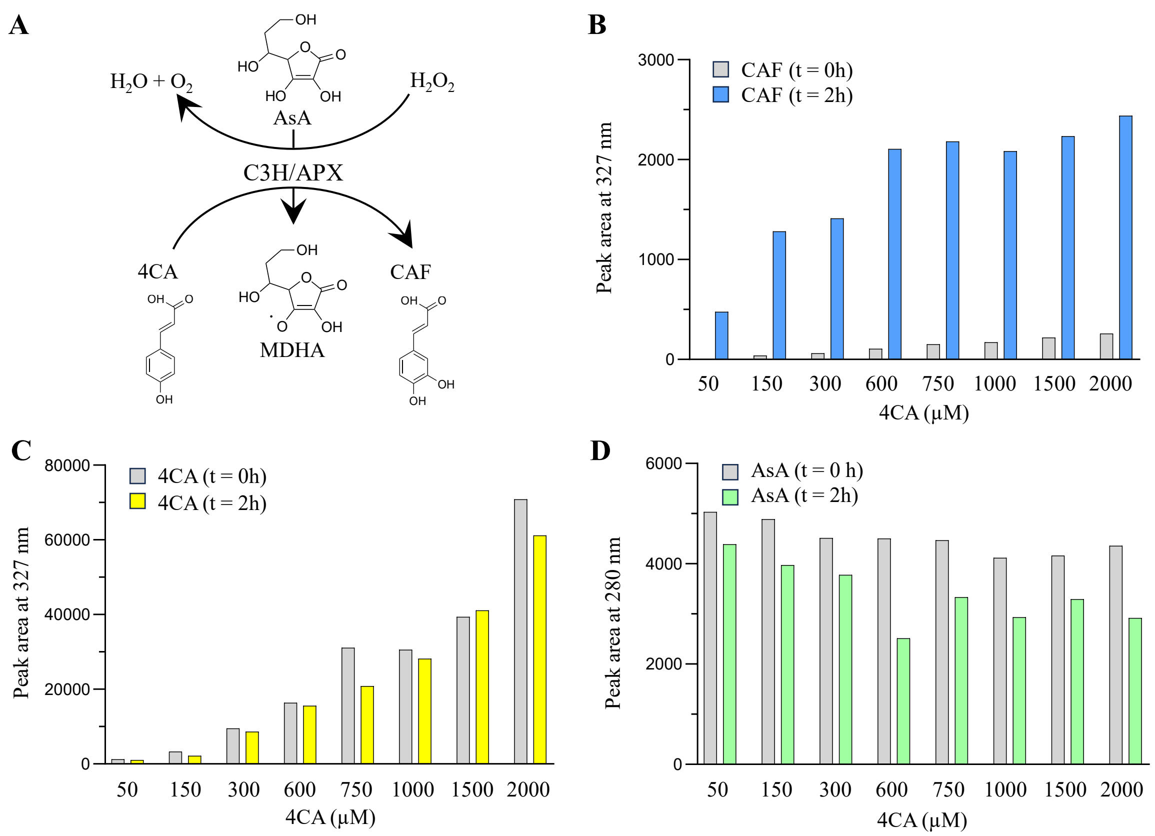

Supplementary Figure S2. C3H/APX catalyzes the enzymatic hydroxylation of 4-coumarate (4CA) to caffeate (CAF) using ascorbate (AsA) as electron donor. (A) Schematic of the bifunctional activity of C3H/APX, showing the coupled redox conversion of AsA to monodehydroascorbate (MDHA) and 4CA to CAF, with H_2O_2 as a substrate. **(B)** Quantification of CAF production over 2 hours across a range of 4CA concentrations (50–2000 μM), as measured by HPLC absorbance at 327 nm. **(C)** Quantification of remaining 4CA after 2 hours, also detected at 327 nm. **(D)** Measurement of ASC consumption after 2 hours of reaction at 280 nm.

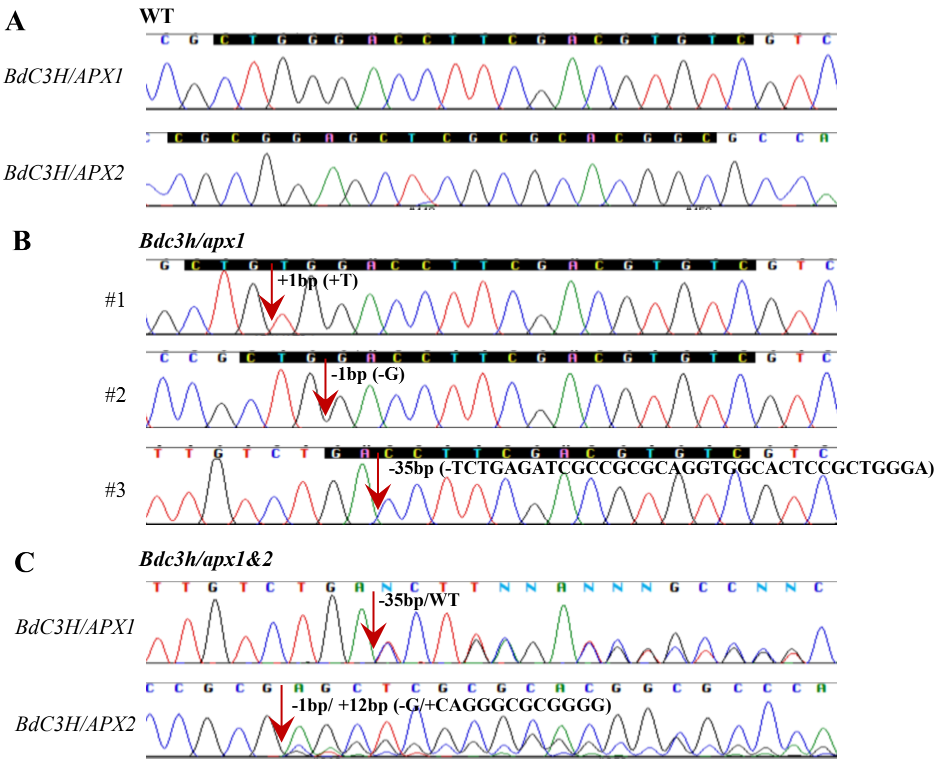

Supplementary Figure S3. Sequencing results of *BdC3H/APXs* gene edited lines. (**A**) Sanger sequencing of target locations for wild type plants. (**B**) Three indel patterns of the *Bdc3h/apx1* plants. (**C**) Sequence traces of *BdC3H/APX1* (*Bradi1g65820*) and *BdC3H/APX2* (*Bradi1g16510*) genes in double *Bdc3h/apx1&2* plants. Red color represents the gene editing location.

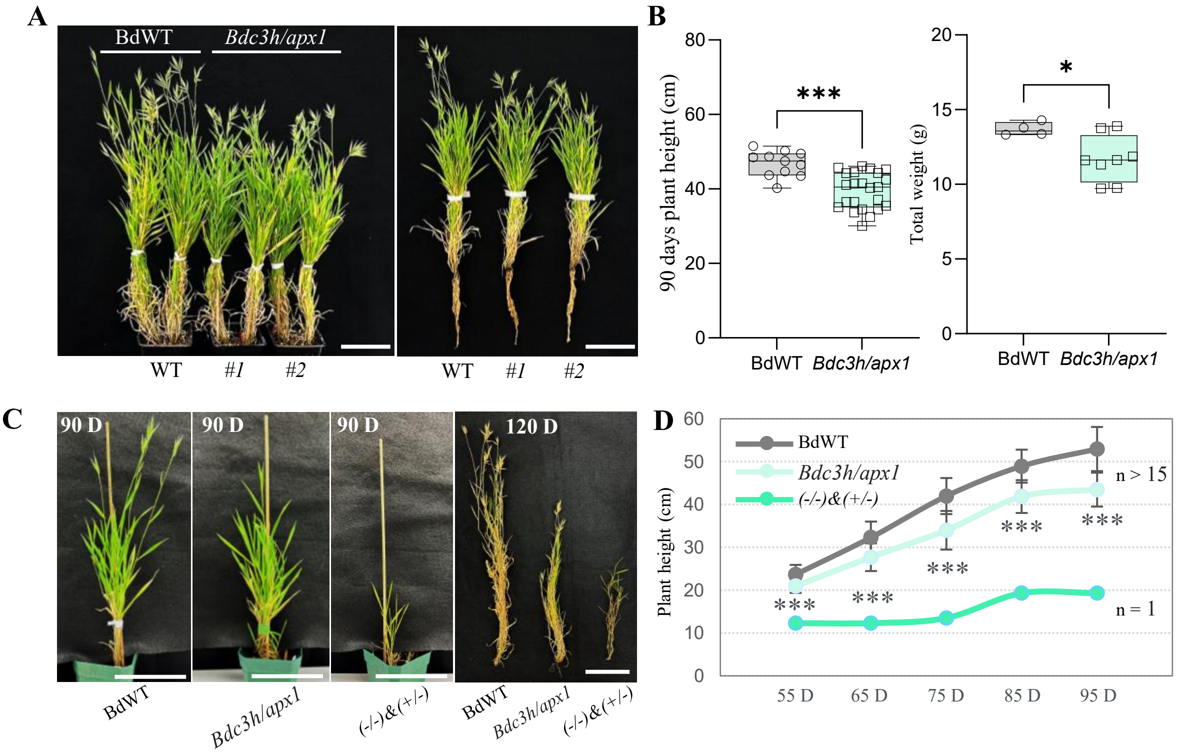

Supplementary Figure S4. Plant growth phenotype of *Bdc3h/apx* plants. (**A**) Plant growth phenotype of *Bdc3h/apx1* plants at 90 days after planting. Scale bar = 10 cm. (**B**) Total weight and plant height of the same plants. Box plots indicate the median (center lines), interquartile range (hinges), and whiskers represents min and max values. Data points for all biological replicates are shown. (**C**) Comparison of growth phenotypes of WT, *Bdc3h/apx1,* and *Bdc3h/apx1&2* mutant plants in T_1 generation plants with different editing events. Scale bar = 10 cm. (**D**) Changes in plant height at different developmental stages in T_1 generation (n > 15 plants). Error bars indicate mean ± SD. Statistical analysis was performed using two-sided unpaired *t*-tests: *$P < 0.05$; ***$P < 0.001$.

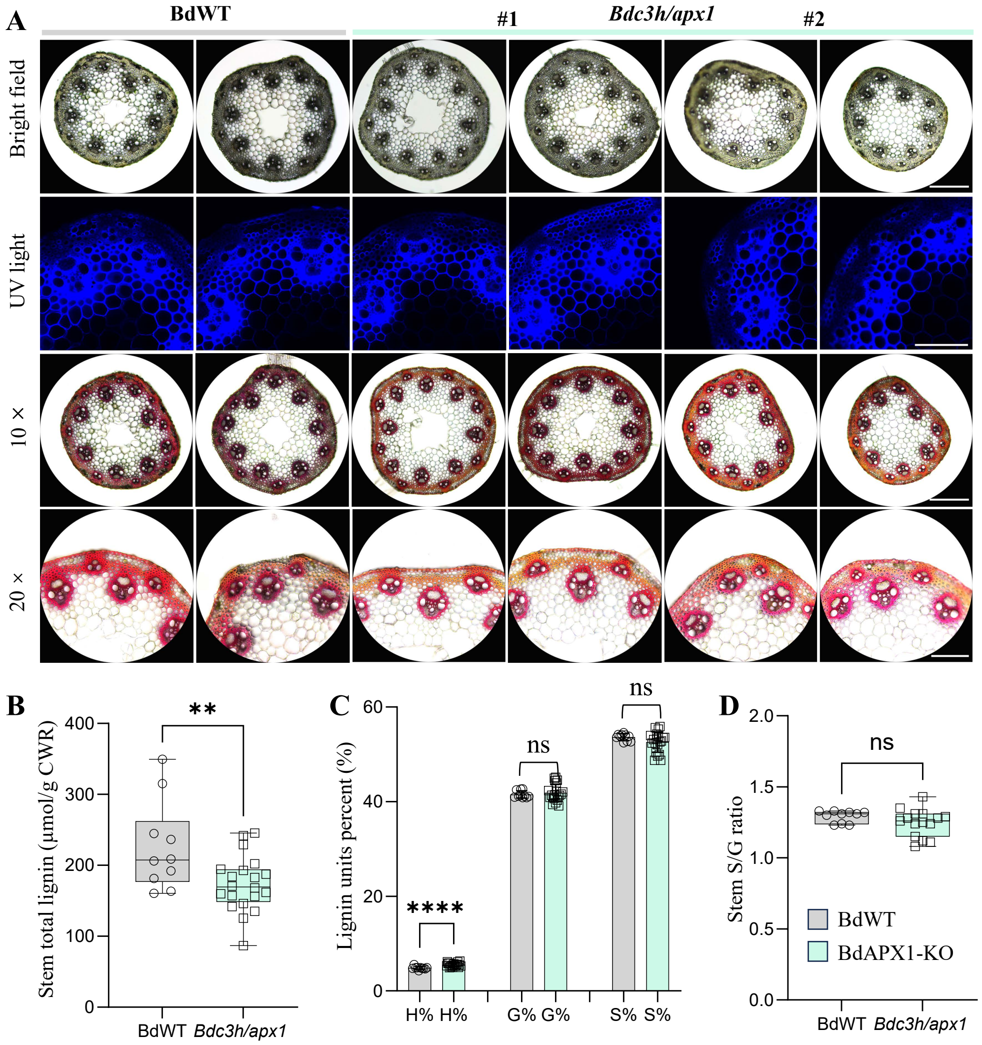

Supplementary Figure S5. Lignin phenotype of *Bdc3h/apx1* plants at 90 days after planting. (**A**) Phloroglucinol-HCl staining results of *Bdc3h/apx1* plants at 90 days. The first row of images shows stem cross-sections under normal light. The second row shows stem cross-sections under UV light. The third and fourth rows show stem cross-sections after staining with Phloroglucinol-HCl. Scale bars: 370 μm for all brightfield and 10× images, and 180 μm for all UV and 20× images. (**B-D**) Stem total lignin (**B**), lignin units percentage (**C**) and S/G ratio (**D**) of *Bdc3h/apx1* and control plants at 90 days after planting. Box plots in **B** and **D** indicate the median (center lines), interquartile range (hinges), and whiskers represents min and max values. Error bars in panel **C** indicate mean ± SD. Data points for all biological replicates are shown. Statistical analysis was performed using two-sided unpaired *t*-tests: $**P < 0.01$; $****P < 0.0001$; ns, not significant.

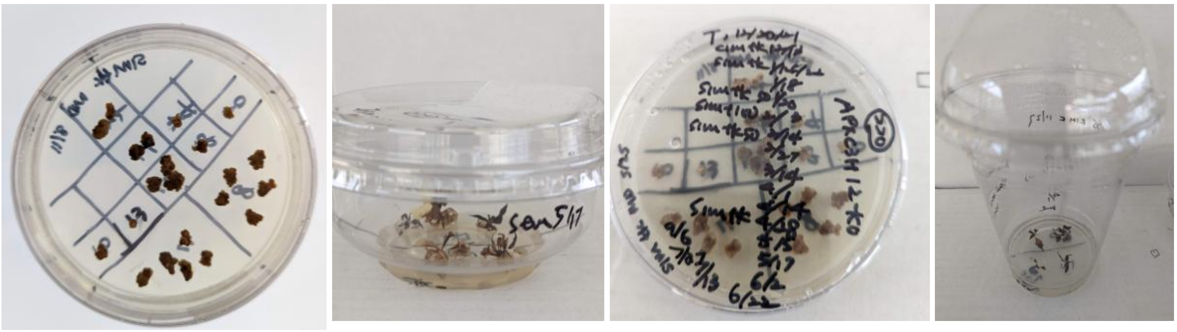

Supplementary Figure S6. Plant phenotype of the *Ptc3h/apx* plants. Despite several attempts, no viable *PtC3H/APX1&2* double genes knockout plants were recovered from shoot induction media in the tissue culture.

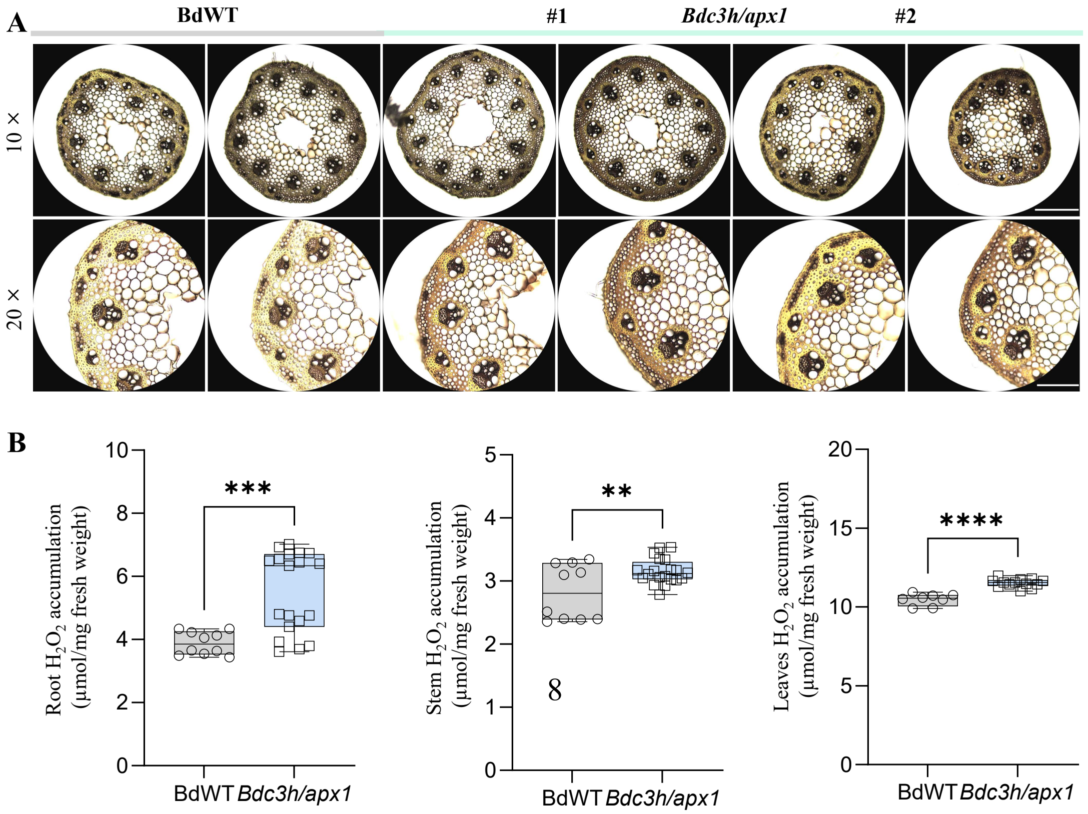

Supplementary Figure S7. H_2O_2 accumulation in different tissues of Brachypodium wild-type (BdWT) and *Bdc3h/apx1* mutants at 90 days after planting. (**A**) DAB (diaminobenzidine) staining showing increased H_2O_2 accumulation in the *Bdc3h/apx1* mutants compared to wild-type plants. Scale bars: 370 µm for all 10× images, and 180 µm for all 20× images. Several of the stem tissue sections shown in panel A are the same as those shown in Figure S5A. (**B**) Quantification of H_2O_2 levels in different tissues using the Amplex Red assay. Box plots indicate the median (center lines), interquartile range (hinges), and whiskers represents min and max values. Data points for all biological replicates are shown. Statistical analysis was performed using two-sided unpaired *t*-tests: $^{**}P < 0.01$; $^{***}P < 0.001$; $^{****}P < 0.0001$.

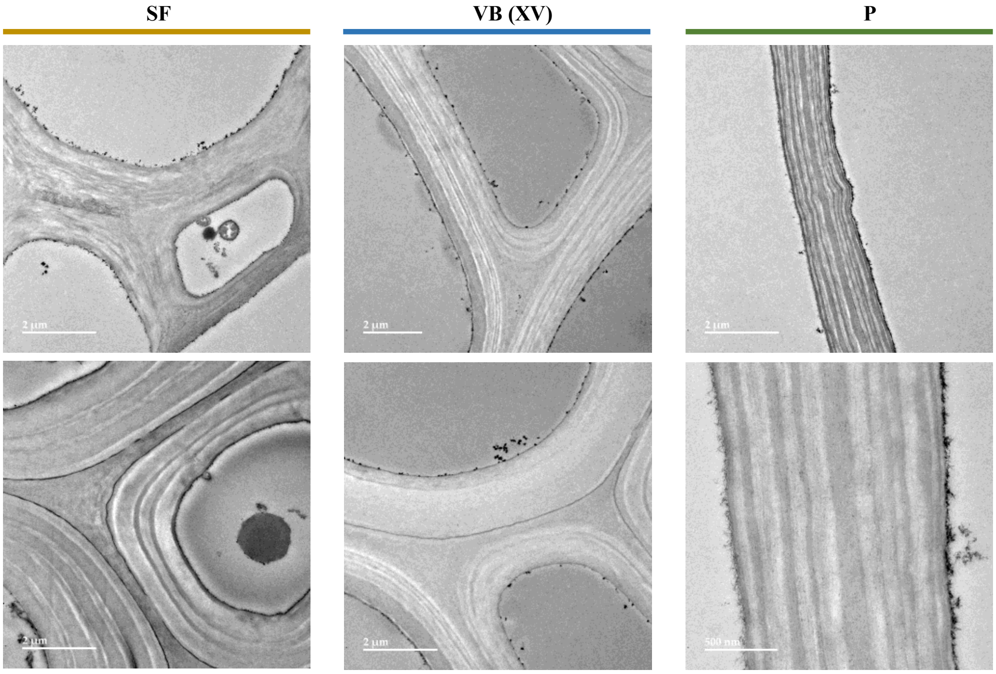

Supplementary Figure S8. Subcellular localization of H_2O_2 in Brachypodium wild-type stem tissues. Transmission electron microscopy (TEM) micrograph images of sclerenchyma fibers (SF), vascular bundles/xylem vessels (VB/XV), and parenchyma cells from 60-day-old *Brachypodium distachyon* wild-type stems following cerium chloride staining. Black dots are cerium perhydroxide deposits and indicate the presence of H_2O_2. Scale bars: 500 nm (bottom right), 2 µm (all other panels).

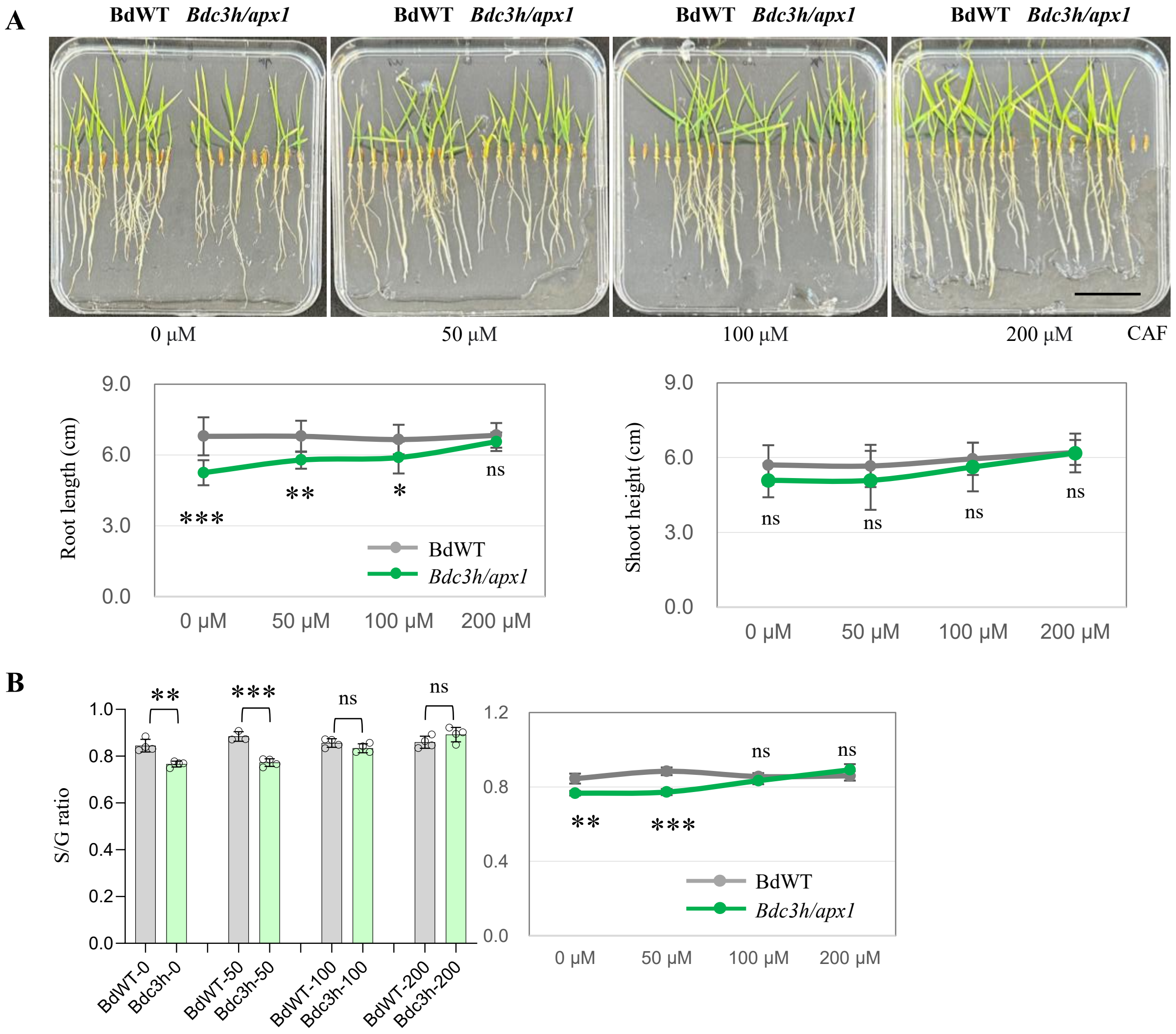

Supplementary Figure S9. Effect of exogenous caffeic acid on growth and lignification of *Bdc3h/apx1* plants. (**A**) Plant growth phenotypes of *Bdc3h/apx1* plants feed with caffeic acid on petri dishes (n = 6-9). Scale bar = 3 cm. (**B**) S/G ratio of caffeic acid fed plants (n = 4). Error bars indicate mean ± SD. Statistical analysis was performed using two-sided unpaired t-tests: *$P < 0.05$; **$P < 0.01$; ***$P < 0.001$; ns, not significant.

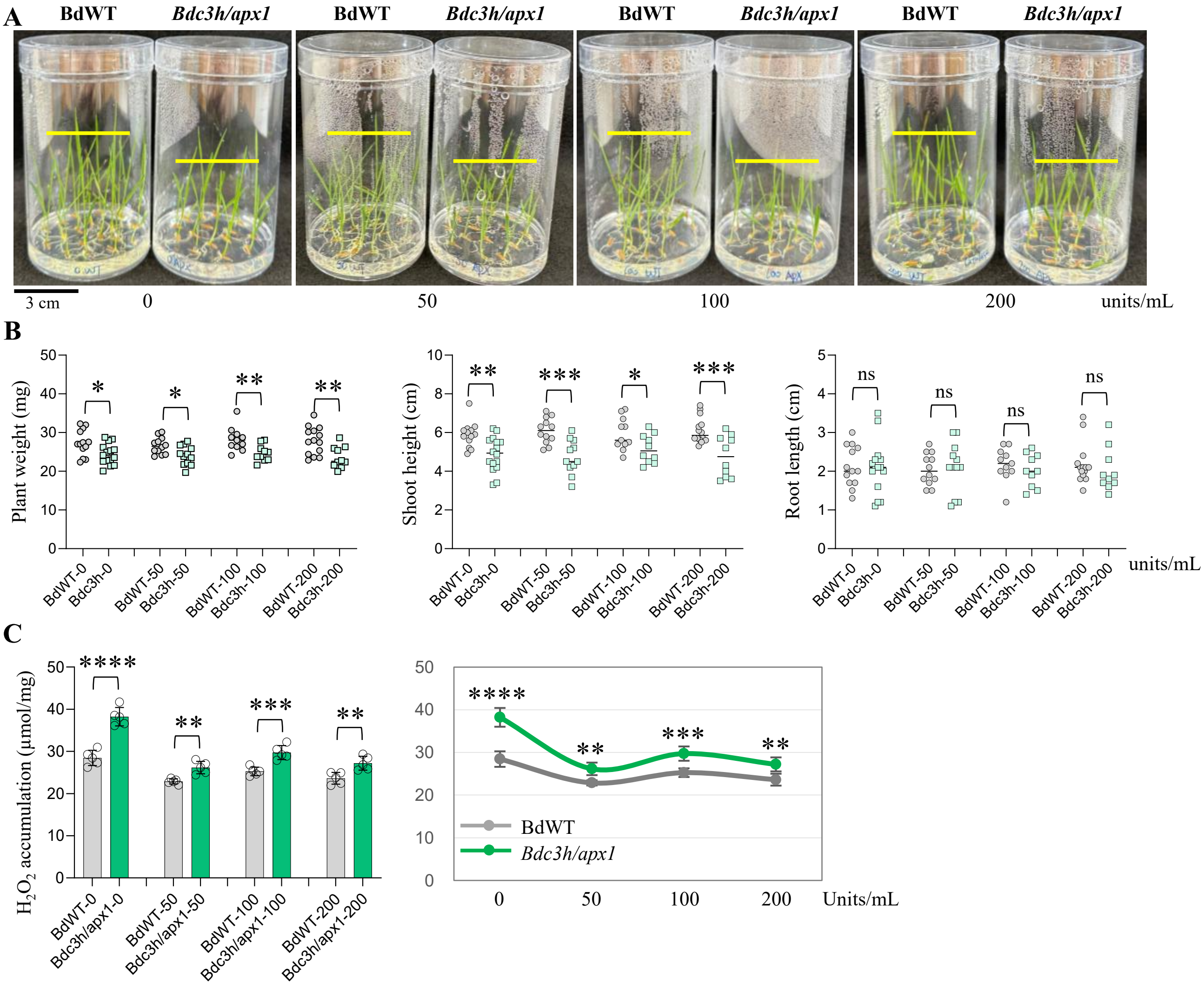

Supplementary Figure S10. Phenotypes of *Bdc3h/apx1* plants feed with catalase. (**A**) Growth phenotype of BdWT and *Bdc3h/apx1* plants treated with exogenous catalase. The yellow horizontal lines represent the height positions of most plants. Scale bar = 3 cm. (**B**) Quantification of plant weight, shoot height, and root length following CAT treatment ($n > 10$). (**C**) H_2O_2 phenotypes of *Bdc3h/apx1* plants feed with catalase ($n = 5$). Data points for all biological replicates are shown. Error bars indicate mean ± SD. Statistical analysis was performed using two-sided unpaired *t*-tests: *$P < 0.05$; **$P < 0.01$; ***$P < 0.001$; ****$P < 0.0001$; ns, not significant.

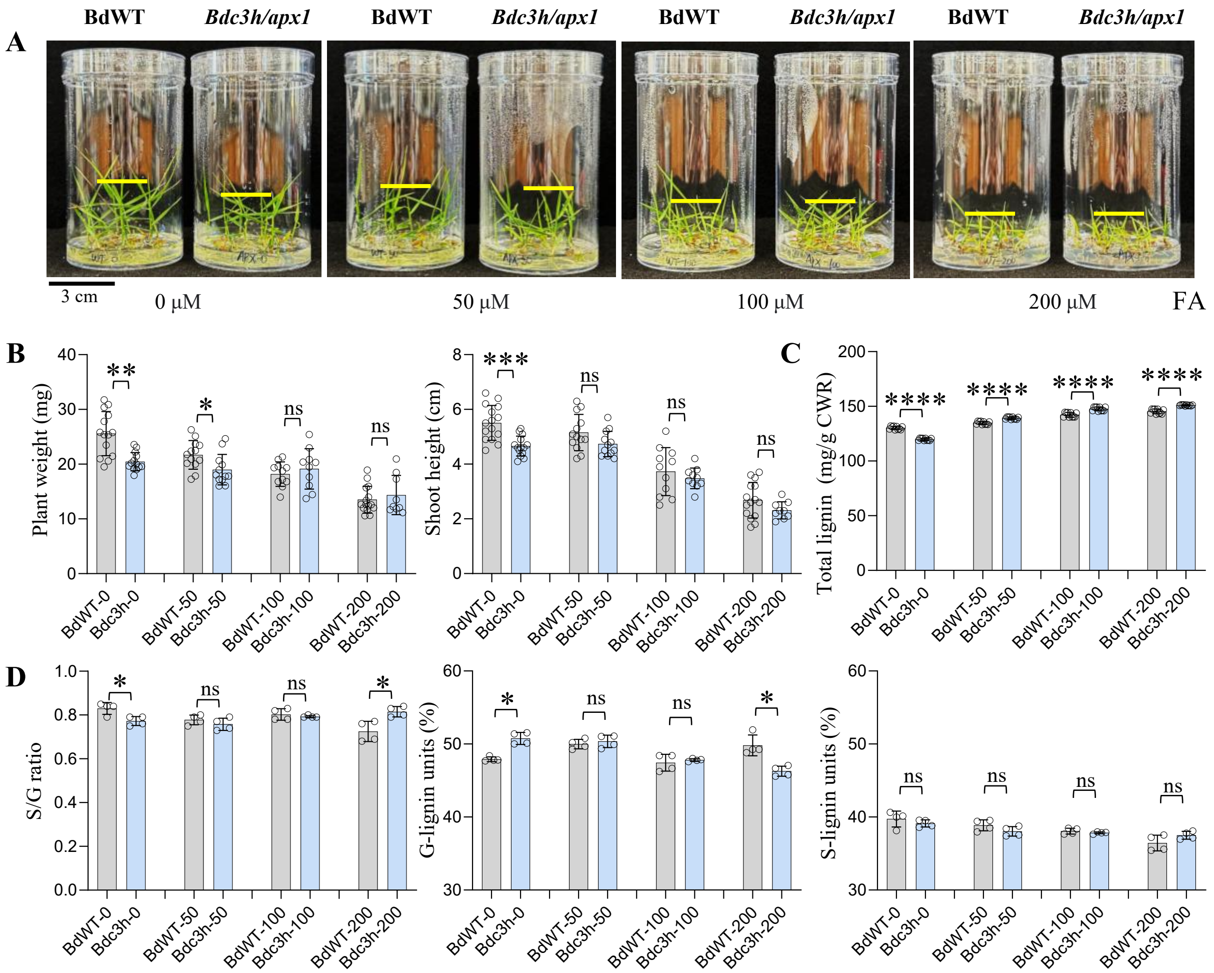

Supplementary Figure S11. Effect of exogenous ferulic acid on growth and lignification of *Bdc3h/apx1* plants. (**A**) Plant growth phenotypes of *Bdc3h/apx1* plants feed with ferulic acid (FA). The yellow horizontal lines represent the height positions of most plants. Scale bar = 3 cm. (**B**) Quantification of plant weight and shoot height of ferulic acid feeding plants ($n > 5$). (**C**) Lignin content following ferulic acid treatment ($n = 5$). (**D**) Lignin composition following ferulic acid treatment ($n = 4$). Error bars indicate mean ± SD. Statistical analysis was performed using two-sided unpaired *t*-tests: *$P < 0.05$; **$P < 0.01$; ***$P < 0.001$; ****$P < 0.0001$; ns, not significant.

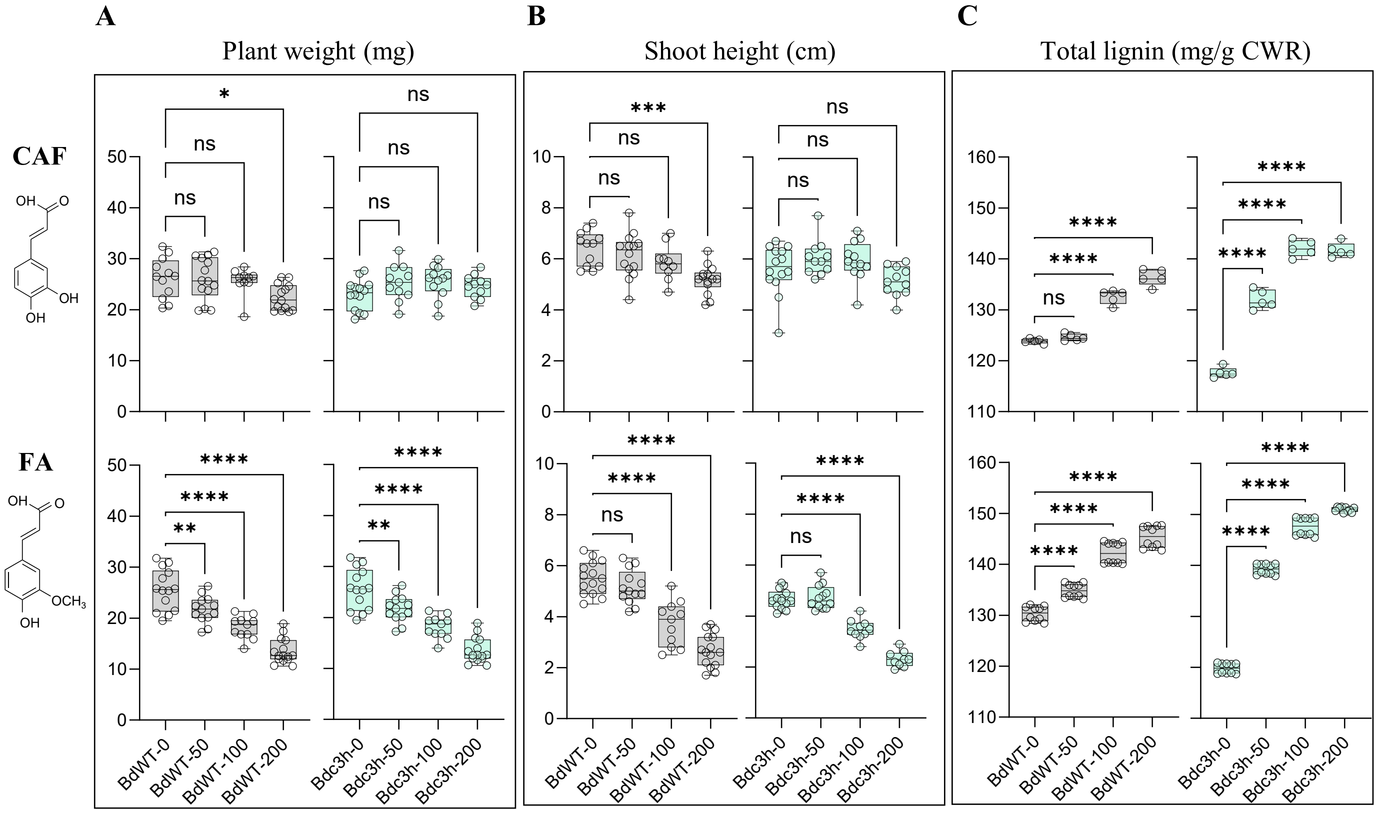

Supplementary Figure S12. Effects of caffeic acid (CAF) and ferulic acid (FA) on plant growth and lignin content in wild type (BdWT) and *Bdc3h/apx1* plants. Plants were grown in culture tubes in MS media with 0, 50, 100, and 200 µM of CAF and FA. Plant weight (**A**) and shoot height (**B**) were measured to assess impact on plant growth, and total lignin content (**C**) was quantified as mg per g cell wall residue (CWR). Boxplots show individual biological replicates. Box plots indicate the median (center lines), interquartile range (hinges), and whiskers represents min and max values. Data points for all biological replicates are shown. Statistical comparisons were performed within each genotype across treatments using one-way ANOVA followed by Dunnett's test for multiple comparisons (*$P < 0.05$; **$P < 0.01$; ***$P < 0.001$; ****$P < 0.0001$; ns, not significant).

Supplementary Table S1. CRISPR/Cas9 mutation patterns of *PtC3H/APX1* alleles in poplar lines.

Cup	Construct	Number of plants	Notes	Pattern	Reduced growth
1	WT717 control	4	control	WT	
2	WT717 control	4	control	WT	
3	WT717 control	3	control	WT	
4	WT717 control	3	control	WT	
5	WT717 control	3	control	WT	
6	Cas9-19 control	3	control	WT	
7	Cas9-19 control	2	control	WT	
8	Cas9-19 control	2	control	WT	
9	Cas9-19 control	3	control	WT	
10	Cas9-19 control	3	control	WT	
11	PtC3H/APX1-KO	4	Monoallelic	-1/WT	
12	PtC3H/APX1-KO	3	Monoallelic	-30/WT	
13	PtC3H/APX1-KO	2	Monoallelic	-30/WT	
14	PtC3H/APX1-KO	5	Monoallelic	+1/WT	
15	PtC3H/APX1-KO	5	Monoallelic	+1/WT	
16	PtC3H/APX1-KO	3	Monoallelic	+1/WT	
17	PtC3H/APX1-KO	5	Monoallelic	+1/WT	
18	PtC3H/APX1-KO	3	Biallelic	NA/-17	*
19	PtC3H/APX1-KO	3	Biallelic	NA/-17	*
20	PtC3H/APX1-KO	2	Biallelic	NA/-17	*
21	PtC3H/APX1-KO	4	Biallelic	+1/-1	*
22	PtC3H/APX1-KO	4	Biallelic	+1 /-1	*
23	PtC3H/APX1-KO	4	Biallelic	+1/-1	*

NA: no-amplification, suggesting large dropouts.

* represents significance difference.

Supplementary Table S2. CRISPR/Cas9 mutation patterns of *PtC3H/APX1&2* alleles in poplar callus lines. All callus lines generated from the *PtC3H/APX1&2* construct exhibited abnormal vascular development during organogenesis and ultimately died (**Fig. S6**)

Cup	Construct	Gene alleles	Pattern
1	PtC3H/APX1&2-KO	PtC3H/APX1a	Indel:+1
		PtC3H/APX1t	Indel:+5
		PtC3H/APX2a	Indel:-2
		PtC3H/APX2t	Indel:-1
2	PtC3H/APX1&2-KO	PtC3H/APX1a	Indel:+1
		PtC3H/APX1t	Indel:+1
		PtC3H/APX2a	Indel:+1
		PtC3H/APX2t	Indel:-1
3	PtC3H/APX1&2-KO	PtC3H/APX1a	Indel:+1
		PtC3H/APX1t	Indel:+1
		PtC3H/APX2a	Indel:-1
		PtC3H/APX2t	Indel:-1
4	PtC3H/APX1&2-KO	PtC3H/APX1a	Indel:+1
		PtC3H/APX1t	Indel:-1
		PtC3H/APX2a	Indel:-1
		PtC3H/APX2t	Indel:-1
5	PtC3H/APX1&2-KO	PtC3H/APX1a	Indel:+1
		PtC3H/APX1t	Indel:+1
		PtC3H/APX2a	Indel:-3
		PtC3H/APX2t	Indel:+1
6	PtC3H/APX1&2-KO	PtC3H/APX1a	Indel:+1
		PtC3H/APX1t	Indel:+1
		PtC3H/APX2a	Indel:-3
		PtC3H/APX2t	Indel:-2
7	PtC3H/APX1&2-KO	PtC3H/APX1a	Indel:-1
		PtC3H/APX1t	Indel:+1
		PtC3H/APX2a	Indel:-1
		PtC3H/APX2t	Indel:-6
8	PtC3H/APX1&2-KO	PtC3H/APX1a	Indel:-1
		PtC3H/APX1t	Indel:+1
		PtC3H/APX2a	Indel:-1
		PtC3H/APX2t	Indel:-2
9	PtC3H/APX1&2-KO	PtC3H/APX1a	Indel:-2
		PtC3H/APX1t	WT_like
		PtC3H/APX2a	Indel:-3
		PtC3H/APX2t	Indel:-1
10	PtC3H/APX1&2-KO	PtC3H/APX1a	Indel:+1
		PtC3H/APX1t	WT_like
		PtC3H/APX2a	Indel:-1
		PtC3H/APX2t	Indel:-1

a: *Populus alba*
t: *Populus tremula*

Supplementary Table S3. Sequences of primers used in this work.

Name	Sequence (5'-3')
PCR Primers:	
BdC3H/APX1-F1	CACAAGCCTTGCTGTCTTCA
BdC3H/APX1-R1	CCTGGGTGAAAGGGGATCT
BdC3H/APX2-F1	TGAACGCTCTTGTTCCTGTC
BdC3H/APX2-R1	GGGCAGGGAAATTGAAAAGT
Restriction sites (for gRNA cloning and genotyping)[a]*:*	
BdC3H/APX1	CCGCTGGGACCTTCGACGTGTC
BdC3H/APX2	CCCGCGGAGCTCGCGCACGGC
gRNA target sequences:	
gBdC3H/APX1-F1	GACACGTCGAAGGTCCCAG
gBdC3H/APX1-R1	CTGGGACCTTCGACGTGTC
gBdC3H/APX2-F1	GCCGTGCGCGAGCTCCGCG
gBdC3H/APX2-R1	CGCGGAGCTCGCGCACGGC
For construct assembly:	
PtC3H/APX1.U6	TCAAGCGAACCAGTAGGCTTGCAAAAACCGGTGGGCCCTT
PtC3H/APX1.SF	AACTTGCTATTTCTAGCTCTAAAACAAGGGCCCACCGGTTTTTGC
PtC3H/APX12:<br>*MTU6.6(tail)-PtC3H/APX1gR*<br>*NA-altSF-linker-*<br>*AtU3d-PtC3H/APX2-SF(tail)*	AAGCGAACCAGTAGGCTTGCAAAAACCGGTGGGCCCTTGTTTCAGA<br>GCTATGCTGGAAACAGCATAGCAAGTTGAAATAAGGCTAGTCCGTT<br>ATCAACTTGAAAAAGTGGCACCGAGTCGGTGCTTTTTTTGCTACCTC<br>AGCATAGTCTACAGCATAAGCTTATGATTTCTTTTTTCTTACGAATT<br>TTGCGTCCCACATCGGTAAGCGAGTGAAGAAATAACTGCTTTATAT<br>ATGGCTACAAAGCACCATTGGTCaCCAAGACAGGAGGGCCATTGTT<br>TTAGAGCTAGAAATAGC
For amplicon sequencing:	
PtC3H/APX(103F)tailR	gttcagacgtgtgctcttccgatCTCATGCTTCGTCTAGCGTATATT
PtC3H/APX(362R)tailF	cctacacgacgctcttccgatcTCAAGGAGTCTGACAGCAATG

Pt, *Populus tremula x alba*; Bd *Brachypodium distachyon*
[a]Underlined regions are restriction sites
[b]Lowercase: Illumina overhangs (binding sites for 2nd barcoding PCR); Uppercase: gene-specific primers.
